# Aesthetic evaluation of body movements shaped by embodied and arts experience: Insights from behaviour and fNIRS

**DOI:** 10.1038/s41598-024-75427-9

**Published:** 2024-10-28

**Authors:** Courtney E. Casale, Ryssa Moffat, Emily S. Cross

**Affiliations:** 1 School of Psychological Sciences, Macquarie University, Sydney, NSW Australia; 2 Professorship for Social Brain Sciences, ETH Zurich, Zurich, Switzerland

**Keywords:** Human behaviour, Social neuroscience

## Abstract

Aesthetic appreciation of full-body movements is likely shaped by our cumulative bodily experiences, yet most of the extant literature in this domain has focused on expertise and familiarity. We ran two experiments exploring individual differences in embodied experience and experience with the arts: In Study 1, we explored how participants’ (n = 41) abilities to learn a choreography shaped their aesthetic perceptions while viewing learned vs. unknown movements, using functional near-infrared spectroscopy (fNIRS) to measure cortical activation over the Action Observation Network (i.e., inferior frontal gyrus [IFG], inferior parietal lobule, middle temporal gyrus [MTG]). Study 1 demonstrated that embodied experience enhanced ratings of enjoyment, familiarity, and reproducibility of movements, and that individual differences in participants’ performance of the learned choreography were not associated with aesthetic ratings, but rather cortical activation in IFG and right MTG while viewing learned choreography. In Study 2, we combined the behavioural data from Study 1 with data from additional participants (total n = 141) to examine the relationship between arts experience and aesthetic perceptions of movements robustly. Study 2 revealed that previous arts and sports experience correlated with aesthetic judgements of familiarity and reproducibility of movements. Our findings highlight the relevance of examining individual experiences to fill theoretical gaps in our understanding of action aesthetics.

## Introduction

Our lived experiences shape our aesthetic landscape^1^. One robust form of evidence for this is that extensive bodily familiarity with movements makes movements more enjoyable to watch^2,3^. Following in this logic, one could propose that only trained ballet dancers stand a chance of appreciating ballet or that only trained actors enjoy watching plays or films. These propositions are quite obviously false! Ballets such as *The Nutcracker* and *Romeo and Juliet* have enthralled expert and non-expert audiences alike for generations, as engaging with art elicits a universal experience. This aesthetic experience is often described as holistic perception, involving brain networks that process sensory–motor, emotion–valuation, and knowledge–meaning information. Together these three networks have been termed the Aesthetic Triad model^4^. Indeed, Blacking^1^ advocates for the importance of zooming out from the stimulus, here dance or theatrical performances themselves, to focus on the sociological and anthropological implications of the arts more globally as an influential form of non-verbal communication.

Recent work demonstrates that arts exposure is relevant, and that details such as the culture in which this exposure was embedded can shape our aesthetic appreciation of movements^5^. Questions remain, however, concerning whether other forms of experience might shape our aesthetic perceptions of body movements. In the present study, we take a first step in exploring the extent to which an observer’s cumulative experience with dance and other related activities shapes their aesthetic preferences for full-body movements. To do so, we query participants’ involvement with and enjoyment of art forms such as theatre, music, painting, dance (in real-life and on social media), as well as sports.

### Repeated exposure enhances aesthetic appreciation of body movements

The two most prominent theories explaining aesthetic appreciation of movements performed by fellow humans hinge on the repeated exposure to body movement^6^. The most prominent, the embodied simulation account, posits that one’s body and prior bodily experiences and expertise shape preferences when observing sequences of body movements (reviewed by Kirsch et al.^7^). Past research has shown increased liking of movements associated with greater embodied experience or expertise^2,5,8,9^, whereby participants reported greater enjoyment of movements either following training^2,8^ or with increasing dance experience acquired over the lifespan^5,9^. Increased liking has also been found to correlate positively with people’s ratings of how well they could reproduce a movement–i.e., a self-report proxy for embodiment of movements^2,5^ (Table 1). These findings suggest a relationship between embodied experience, perceived reproducibility, and liking of watched movements. However, two studies found that non-dancers preferred *less* reproducible movements^10,11^, which the authors attribute to a preference for greater kinematic variability and complexity^11^. Considered together, the empirical evidence collected to date suggests an important modulatory influence of embodied experience on the subjective evaluation of dance movements.

**Table 1:** Definitions of the dependent variables explored in this study. They are each relevant at the level of the individual (i.e., a participant’s embodied experience, a participant’s self-reported embodiment, a participant’s performance ability, a participant’s engagement with arts and sports).

Participants’	Variable type	Conceptualisation
Self-reported enjoyment	Continuous	Aesthetic judgement The extent to which participants enjoyed observing movement.
Self-reported visual experience	Continuous	Aesthetic judgement reflecting visual fluency with movements. The extent to which participants perceive movements as being familiar.
Self-reported embodiment	Continuous	Aesthetic judgement reflecting embodiment of movements. The extent to which participants perceive the movements as being reproducible with their own body.
Embodied experience	Categorical	Experimental manipulation differentiating between movement sequences that participants did and did not physically perform. Performed movements are part of participants’ embodied experience, while movements that they did not perform are not.
Performance ability	Continuous	Score of how well movements have been learned. The extent to which the embodied experience gained through physically performing dance movements, during the training component of the study, is evident to an outside observer.
Engagement with arts and sports	Categorical/Continuous	Self-report measure of experience with performing arts. A) Do participants have experience with different arts and sports? B) The extent to which participants enjoy various arts and sports.

The second most prominent theory, the fluency of processing theory, offers another potential explanation. This theory posits that the ease with which one’s brain processes information, such as appreciation of movement sequences, increases as one becomes more familiar with the information^3,12,13^, and one may even tend to reject unfamiliar movement repertoires at first. Accordingly, Orgs et al.^14^ revealed that observers prefer stimuli rated as more familiar (i.e., a self-report proxy for visual experience; Table 1), suggesting an increase in the ease of processing, and subsequent likability, via an exposure effect. For example, in 1913, audiences rejected Stravinsky’s unconventional *Rite of Spring* when it first premiered, as they had previously only been exposed to classical ballet^3,15^. Over time, though, modern choreographies became more commonplace, and audiences began to enjoy such “odd” movements increasingly so because these movements were gradually incorporated into their knowledge base^3,16^. The familiarity and fluency-based explanation of aesthetic appreciation provides a framework for the interaction between embodied experience or expertise and visually acquired kinematic knowledge in aesthetic evaluations of body movements^17^.

### Individual differences in experience and enjoyment of the arts

In addition to a common prerequisite, i.e., repeated exposure to body movements, these theories also draw from the same pool of empirical evidence: Most studies operationalise dance experience, whether gained over a lifetime or over a short training session, to manipulate embodied experience (Table 1). There are, of course, other avenues beyond dance through which one can gain exposure to communicative full-body movements. While everyday social interaction may come to mind first, we are particularly interested how individuals’ experience with other art forms shapes their aesthetic perceptions of human movement. Individuals involved in artistic or movement-based media tend to engage in other forms of art and/or movement as well^18,19^, which raises the question as to whether people who seek arts experiences are more acutely attuned to and/or appreciative of full-body movements than those who engage less with art. Thus, to broaden our understanding of the extent to which experience with the arts, beyond dance, influences aesthetic perceptions of body movements, we focus on how individual differences in experience with and enjoyment of visual, musical, or dramatic arts influence enjoyment of body movements (Table 1).

Recent work comparing aesthetic appreciation between dance movements from one’s own culture and those from other cultures demonstrated that inexperienced viewers of art and dance report a strong preference for art from their own culture, while previous experiences may foster appreciation of art from another culture^5,20^. Accordingly, an individual’s previous engagement with arts and movement activities can also influence preferences^21,22^. Indeed, Sevdalis & Raab^23^ found that the amount of sports training correlated with accuracy in identifying a dancer’s intended intensity of expression, with greater sports engagement predicting higher identification accuracy. Survey-based studies also indicate a tendency for people interested in one art form to engage in other art forms^18,19^. Finally, individual differences in the ability to learn and perform movements have also been demonstrated to impact aesthetic appreciation, with better learning and performance corresponding to greater appreciation^2,24–26^. Stemming from the embodied simulation account of aesthetics^7,27^, performance ability represents the degree to which an outside observer sees physical evidence of embodiment of specific movements (Table 1).

### The present pair of studies

We present here our findings from two preregistered studies (Study 1: https://osf.io/cndfg/; Study 2: https://osf.io/vfsn2/). In Study 1, we examine the extent to which gaining embodied dance experience (i.e., learning a choreography) impacts ratings of enjoyment, familiarity, and reproducibility when viewing learned and unknown body movements. In Study 1, we also explore the degree to which participants’ ability to perform a learned choreography and aesthetic ratings correlate with cortical responses to learned and unknown choreographies.

Participants (n = 41) first completed questionnaires querying their experience and enjoyment of several types of art (painting, music, theatre, dance). Second, participants rated videos of short sequences of body movements (i.e., dance) for enjoyment, familiarity, and reproducibility of the movements. Third, participants learned a short choreography composed of half of the previously rated movement sequences. Next, participants repeated the same rating task again while we recorded cortical activation over three bilateral action observation network (AON) regions (the inferior frontal gyri [IFG], the middle temporal gyri [MTG], and the inferior parietal lobules [IPL]) using fNIRS. We selected fNIRS for its strong temporal resolution and low sensitivity to participant movement, relative to other functional neuroimaging techniques^28^. Moreover, recent work demonstrates the utility of fNIRS in examining the role of the AON in aesthetic judgements of gymnastic movements^29^. Combining these behavioural and cortical measures, Study 1 assesses how embodied experience gained by learning a choreography, and individuals’ abilities to perform a learned choreography influence aesthetic perceptions and processing at a cortical level.

Midway through data collection for Study 1, we determined that additional data would be required to address questions pertaining to individual differences in arts experience and aesthetic judgements in an empirically responsible way. We thus collected data from 100 additional participants who only judged movements once and did not undergo dance training or brain imaging, with the intention to aggregate the matching behavioural data set. In Study 2, with the aggregated data (n = 141), we explore the influence of previous arts and sports experience on aesthetic perceptions of dance choreography.

In summary, the present work bridges existing theories and recent calls to consider individual observers’ lived experiences using individual experience and enjoyment of the arts, ability to learn a choreography, as well as self-reported familiarity and reproducibility. This combination of measures positions the present study to contribute meaningfully to our theoretical understanding of several facets of embodiment in action aesthetics.

## Results

### Study 1 – Behavioural and cortical correlates of embodied dance experience

In Study 1, as per our preregistration, we first compared behavioural perceptions of enjoyment, familiarity, and reproducibility of body movements, before and after participants learned half of these movements. The learned movements can be said to be “embodied” by participants. Next, we examined the influence of participants’ individual abilities to perform the choreography on their aesthetic ratings and cortical responses to the movement sequences they learned vs. similar, but unknown sequences.

Note: As preregistered, we also assessed cortical activation evoked by learned and unlearned movements in AON regions, as a replication of previous fMRI work with fNIRS. We report this analysis in the Supplementary Materials (Figure S2, Tables S2–S5), and in the main text, we direct our attention toward how individual differences shape aesthetic processing and perception.

#### Aesthetic ratings before and after learning a choreography

**Fig. 1:**
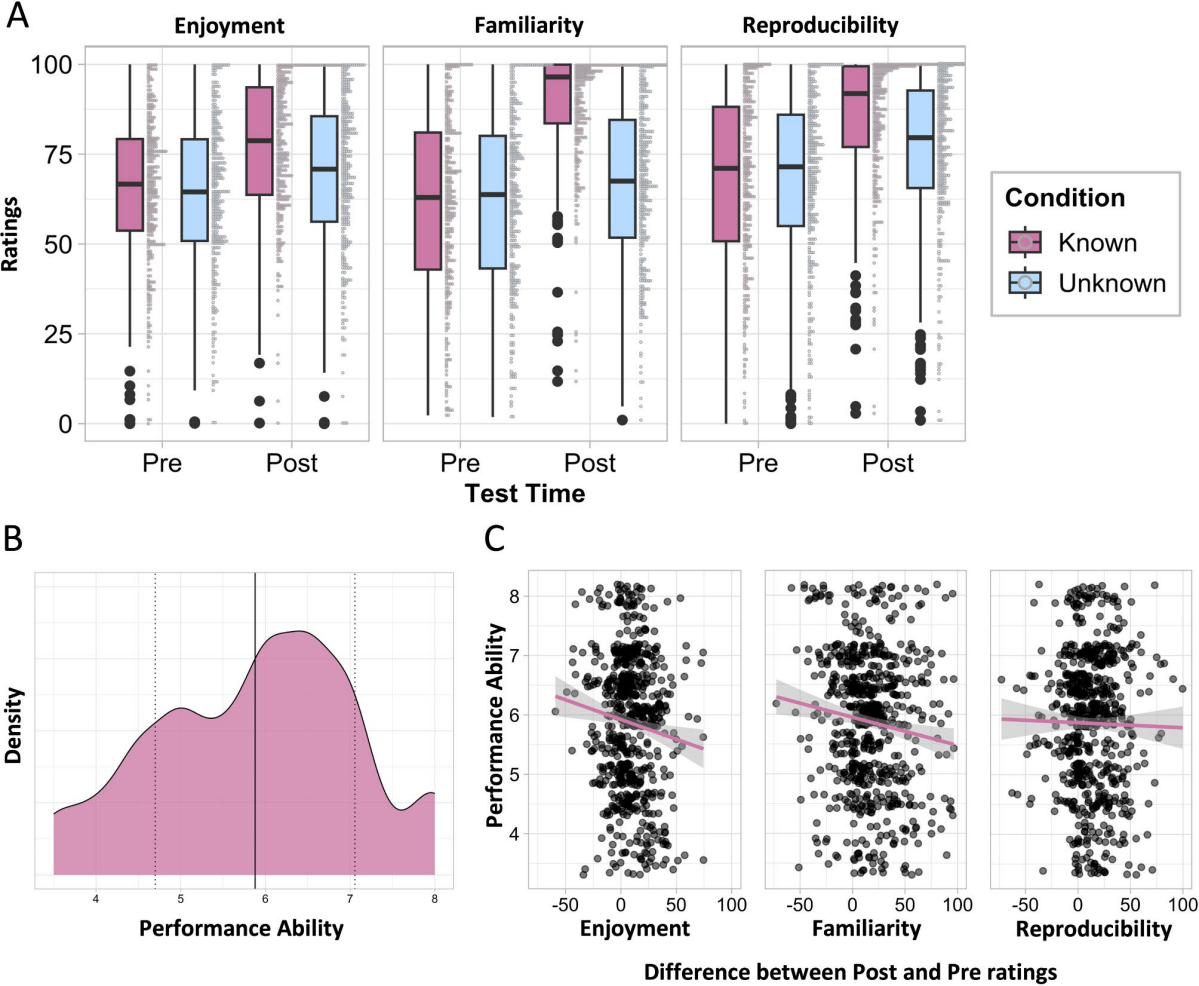
(**A**) Ratings of enjoyment, familiarity, and reproducibility for known (pink) and unknown (blue) movements before (Pre) and after (Post) learning a choreography. (**B**) Distribution of participants’ ability to perform the learned choreography with mean (solid line) and ± 1 standard deviation (dashed lines). (**C**) Relationships between participants’ performance ability and ratings of enjoyment, familiarity, and reproducibility of the movement sequences (note: x-axis represents post–pre training difference in ratings).

To assess differences in aesthetic ratings before and after dance training, we modelled each aesthetic measure separately (see "Group-level analyses–Behavioural responses and cortical activity" for detailed description of model selection process). For enjoyment the best model was *enjoyment* ~ *1* + condition*testTime + *(1|ID),* for familiarity: *familiarity* ~ *1* + condition*testTime + *(1|ID),* and for reproducibility: *reproducibility* ~ *1* + condition*testTime + *(1* + *condition* + *testTime|ID).* Before participants learned a choreography, their (“Pre”) ratings for all three aesthetic measures were similar in known and unknown conditions (i.e., no significant differences, all *p*s > 0.05; Fig. 1A, Table S1 in Supplementary Materials). After learning a choreography (“Post”), participants rated all movement sequences (those that they had learned and those that they had not) as more enjoyable (*ß* = 4.73, CI = [2.46, 7.01], *p* =  < 0.001), familiar (*ß* = 10.84, CI = [8.23, 13.44], *p* =  < 0.001), and reproducible (*ß* = 4.62, CI = [0.89, 8.35], *p* = 0.003) than before (Fig. 1A, Table S1). Participants further rated movements they learned during the training, compared to unlearned movements, as more enjoyable (*ß* = 7.93, CI = [4.71, 11.15], *p* =  < 0.001), more familiar (*ß* = 22.53, CI = [18.84, 26.22], *p* =  < 0.001), and more reproducible (*ß* = 9.48, CI = [4.96, 13.99], *p* =  < 0.001; Fig. 1A, Table S1). This difference in ratings between before and after learning a choreography suggests that embodied experience may exert a positive influence on aesthetic perceptions of full-body movements.

#### Individual differences in performance of learned choreography

Next, we examined the influence of individual differences in performance ability on aesthetic ratings. To do so, we calculated the difference between each participant’s ratings before (“Pre”) and after (“Post”) dance training to be employed as the outcome variable in our models. Participants’ performance of the choreography was rated from 1 to 10 by two dance experts (mean = 5.88, SD = 1.19; Fig. 1B; scoring procedure described in "Dance training and assessment of performance ability"). For each measure (fit separately) we fit the following model: *pre-post difference in enjoyment/familiarity/reproducibility* ~ *1* + *performance* + *condition* + *(1* + *condition|ID)*. We observed no significant relationship between ability and any aesthetic of the three ratings (Fig. 1C; Table S6 in Supplementary Materials; all *p*s > 0.05).

During the review process, we also examined the relationship between participants’ performance scores and their aesthetic ratings before and after learning the choreography (i.e., not as a difference score). Participants’ performance scores did not predict participants’ ratings of enjoyment or reproducibility (all *p*s > 0.05). For ratings of familiarity, better performance scores were associated with lower ratings of familiarity for unknown movement after training (*ß* =  − 3.91, CI = [− 7.50, − 0.31], *p* = 0.033). Performance scores did not predict ratings of familiarity before training or for known movements after training (*p*s > 0.05).

#### Unique contributions of viewing and evaluating movement to cortical activation

**Fig. 2:**
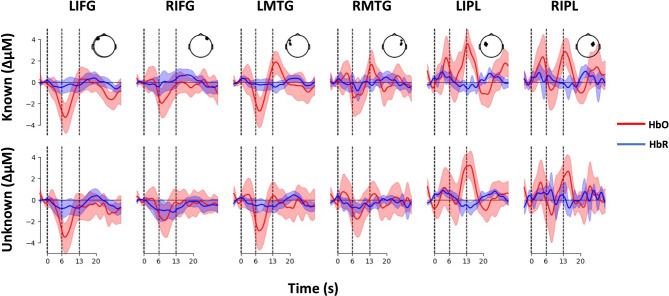
Waveform of cortical activation in bilateral inferior frontal gyrus (IFG), middle temporal gyrus (MTG), and inferior parietal lobule (IPL) measured using fNIRS. HbO = oxygenated haemoglobin; HbR = deoxygenated haemoglobin. Dotted vertical lines indicate 6 s (end of video viewing) and 13 s (end of viewing and responding to aesthetic judgment), respectively. Inset head diagrams show optode location for each ROI. Shaded areas along the waveform represent the 95% confidence interval.

Next, we sought to examine the cortical activation arising from differences in performance ability. That is, we focused on the extent to which individuals’ ability to perform the choreography predicted their cortical activity while viewing and judging movement sequences. To do this, we recorded cortical activation using fNIRS over regions of interest (ROIs) associated with the AON while participants viewed movement sequences (known and unknown in a random order) and rated them for enjoyability, familiarity, and reproducibility (Fig. 6). During visual inspection of the waveforms (Fig. 2), we expected to see a positive peak in HbO, with a negative peak in HbR, at approximately 6 s post-video-onset, with the signal then returning to baseline with no further notable deviations. Instead, we observed an unexpected negative peak in HbO at ~ 6–7 s post-video-onset and a later positive peak at ~ 13 s post-video-onset. After careful consideration of the design of our experiment (see Methods section), we propose that the first negative peak aligns with the viewing of the video and the second peak aligns with the explicitly evaluative stage of responding to an aesthetic rating question. Exploratorily, we opted to fit two separate generalised linear models (GLMs), thereby estimating the amplitude of haemodynamic responses for each peak and gaining separate insight into the processes of viewing body movements (involving spontaneous aesthetic evaluation) and rating the movements for their aesthetic value (explicit consideration of the aesthetic evaluation). Further details about recording and analysis procedure are found in the Methods sections: "fNIRS equipment and optode positioning–Group-level analyses–Behavioural responses and cortical activity". Our preregistered analyses replicating previous fMRI work with fNIRS (contrasting cortical activation for known vs. unknown) are reported and discussed in detail in Supplementary Material (pages 1–4; Study 1).

#### Performance ability and cortical activation

**Fig. 3:**
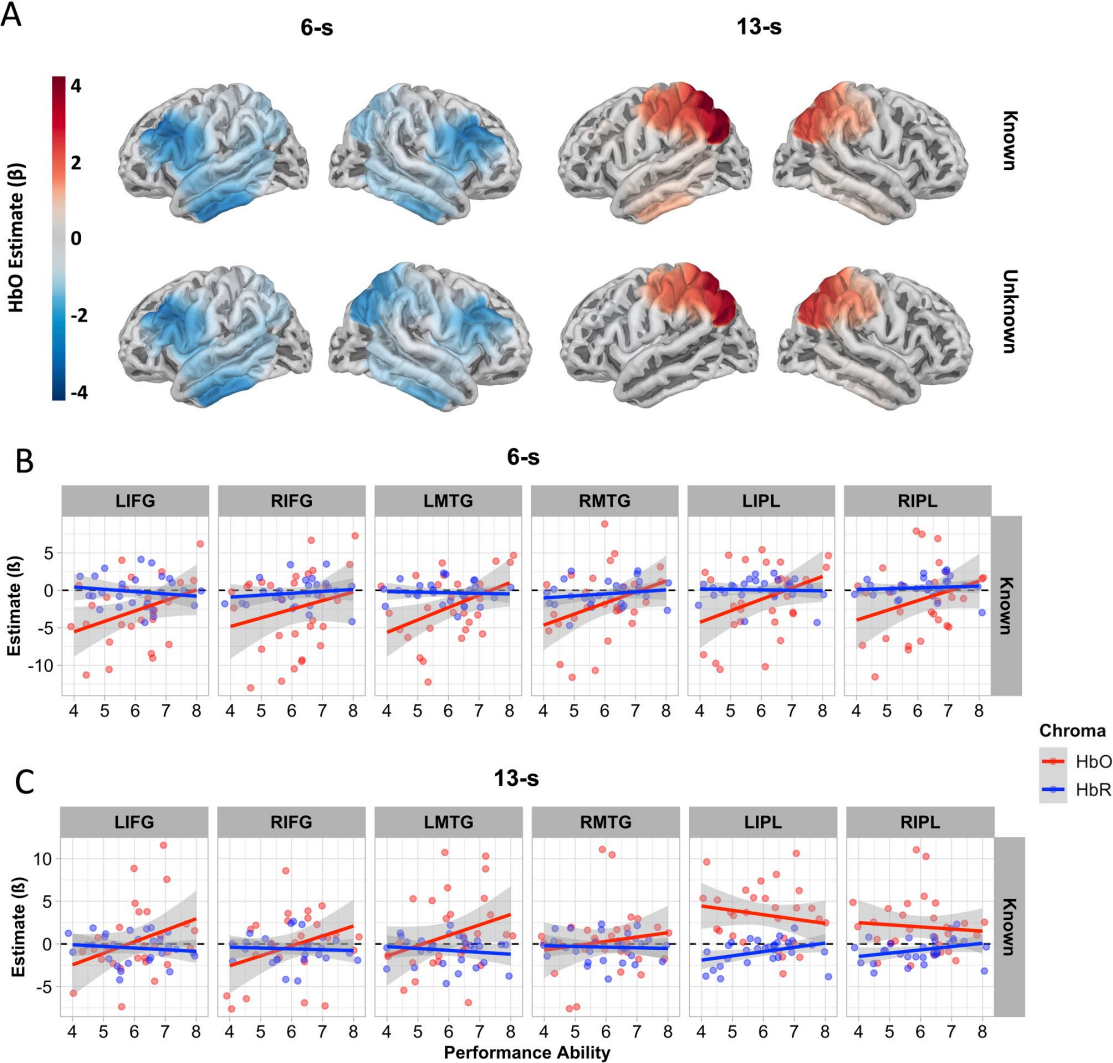
(**A**) Estimates of cortical activation from GLM analysis projected onto brain surface (HbO only) using 6-s and 13-s boxcar models. (**B**) Relationship between performance ability and cortical activation while viewing videos (6-s boxcar) per ROI and chromophore for the known condition only. (**C**) Relationship between performance ability and cortical activation while viewing videos (13-s boxcar) per ROI and chromophore for the known condition only.

GLM analyses with a 6-s boxcar examining the influence of performance ability on cortical activation revealed that HbO concentrations increased with increasing ability in bilateral MTG (left: *ß* = 2.58, CI = [0.59, 4.58], *p* = 0.012; right: *ß* = 2.03, CI = [0.03, 4.02], *p* = 0.047) and right IFG (*ß* = 2.30, CI = [0.31, 4.30], *p* = 0.024; left IFG trending only: *ß* = 1.95, CI = [-0.04, 3.95], *p* = 0.055) when viewing learned movements (Fig. 3, Figure S3, Table S4 in Supplementary Materials). That is, individuals with greater performance ability showed less negative estimates in these regions while viewing movement sequences that they had learned. No significant associations were observed between performance ability and cortical activation using the 13-s boxcar in any ROI (all *p*s > 0.05; Table S5 in Supplementary Materials).

### Study 2 – Individual differences in engagement with the arts and aesthetic perceptions of body movements

In Study 2, we conducted our preregistered analyses to examine whether participants’ previous experience with the arts and enjoyment of the arts and sports impacted aesthetic evaluations of body movements. We also assessed the relationships between participants’ aesthetic judgements in non-preregistered, exploratory analyses. We found that ratings of familiarity correlated positively with ratings of enjoyment (*ß* = 0.17, CI = [0.14, 0.21], *p* < 0.001) and reproducibility (*ß* = 0.07, CI = [0.03, 0.10],* p* = 0.001). Further, we observed that ratings of enjoyment correlated negatively with ratings of reproducibility (*ß* = -0.16, CI = [-0.20, -0.13], *p* < 0.001).

#### Experience with arts and sports

**Fig. 4:**
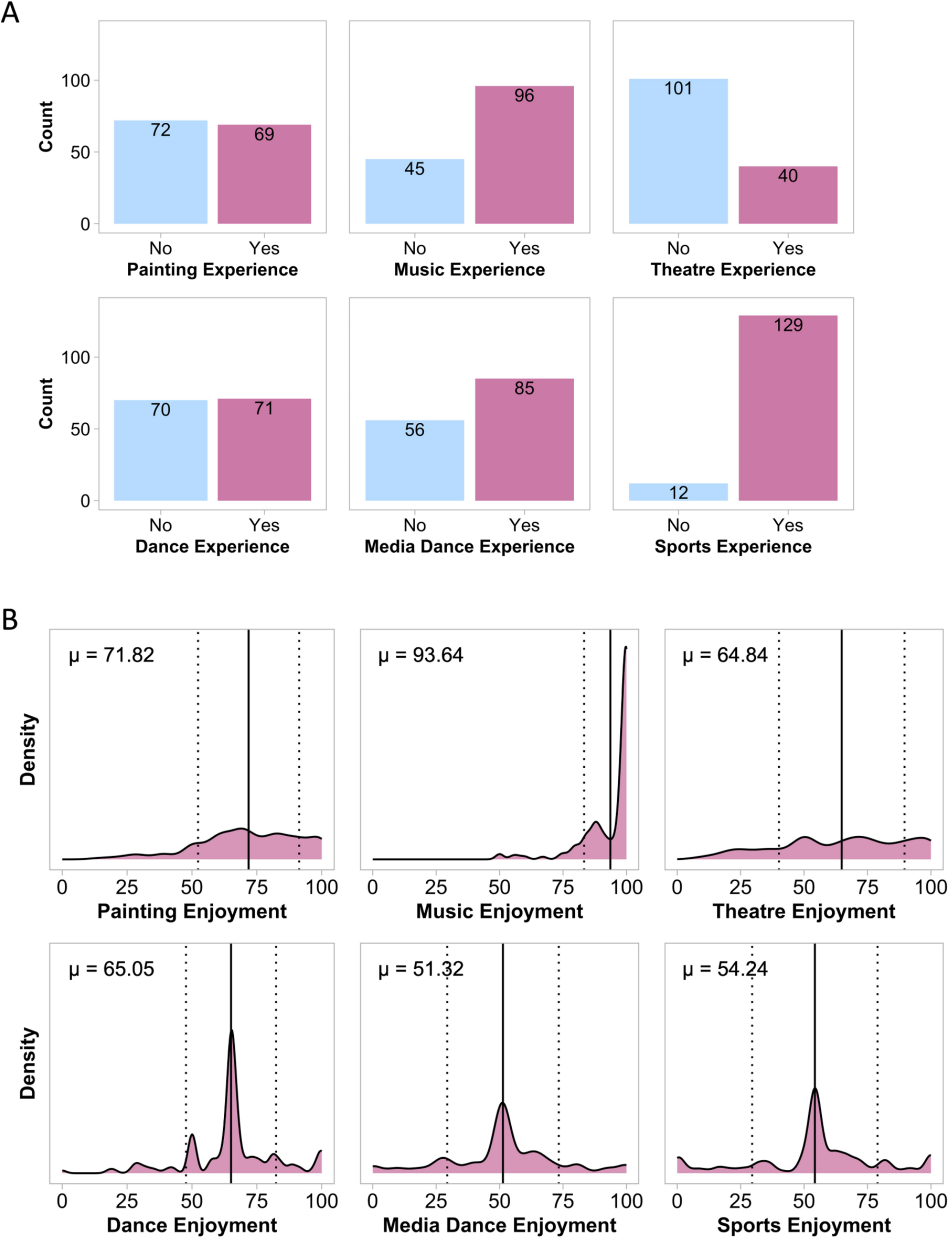
(**A**) Number of participants who reported having experience with painting, music, theatre, dance, social media dance, and sports. (**B**) Distributions of reported enjoyment of painting, music, theatre, dance, social media dance, and sports. µ and solid vertical lines show mean; dashed vertical lines show standard deviation.

Many of our participants reported previous experience with one or more performing art or sport (Fig. 4A). As a note, when indicating the extent of their “experience,” participants were encouraged to consider any experience they had ever had with the arts or sports, including as a spectator. For enjoyment, the best model was *enjoyment* ~ *1* + *(1|ID)*, with the absence of predictor terms indicating that no types of arts or sports experience were predictive of enjoyment ratings. For familiarity, the best model was *familiarity* ~ *1* + *painting_experience* + *dance_experience* + *(1|ID)*. Greater familiarity with the observed body movements was correlated with painting experience (*ß* = 68.2, CI = [64.7, 71.7], *p* =  < 0.001) and dance experience (*ß* = 67.5, CI = [64.1, 70.9], *p* =  < 0.001; Table S7in Supplementary Materials). For reproducibility, the best model was *reproducibility* ~ *1* + *media_dance_contrast* + *(1|ID),* where higher reproducibility ratings were associated with experience watching dance on social media platforms (*ß* = 73.2, CI = [70.0, 76.5], *p* =  < 0.001; Table S7).

#### Enjoyment of arts and sports

**Fig. 5:**
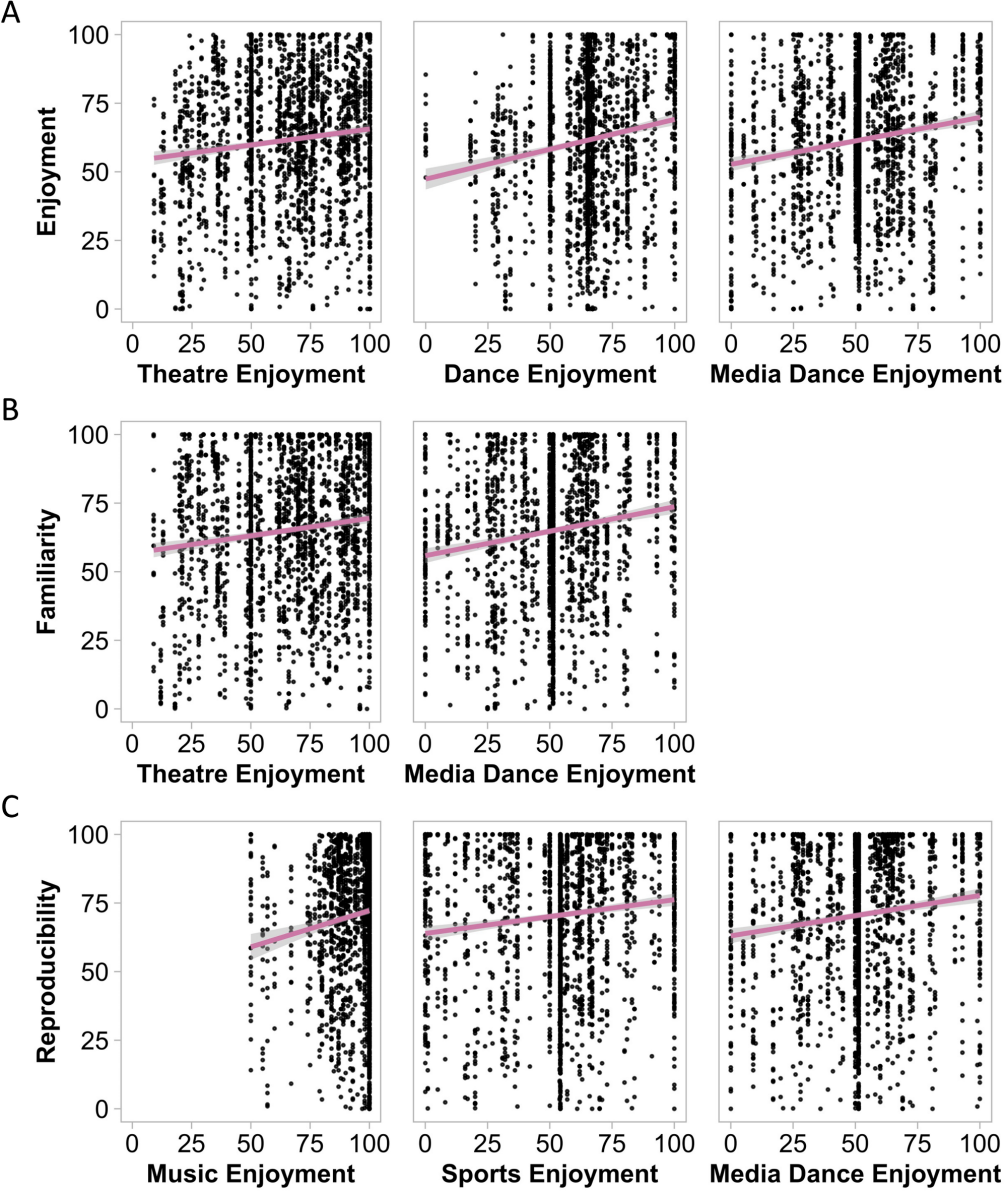
Reported enjoyment of arts types in relation to each (**A**) enjoyment, (**B**) familiarity, and (**C**) reproducibility ratings of dance movements (ß indicated by pink line). The arts types presented for each rating are those which were included in the final models.

Participants generally reported the greatest enjoyment of music, whereas there was more variability for enjoyment of the other art forms and sports (Fig. 4B). For enjoyment, the best model was *enjoyment* ~ *1* + *theatre_enjoyment* + *dance_enjoyment* + *media_dance_enjoyment* + *(1|ID)*, though the enjoyment of these arts and sports was not significantly correlated with enjoyment of the choreography (*p*s > 0.05; Fig. 5; Table S8 in Supplementary Materials). For ratings of familiarity, the best model was *familiarity* ~ *1* + *theatre_enjoyment* + *media_dance_enjoyment* + *(1|ID)*. Greater familiarity of the movements was correlated with greater enjoyment of theatre (*ß* = 0.11, CI = [0.01, 0.21], *p* = 0.024) and social media dances (*ß* = 0.17, CI = [0.06, 0.27], *p* = 0.003). For reproducibility, the best model was *reproducibility* ~ *1* + *music_enjoyment* + *sports_enjoyment* + *(1|ID)*, where greater reproducibility was associated with greater enjoyment of music (*ß* = 0.26, CI = [0.01, 0.50], *p* = 0.038) and sports (*ß* = 0.11, CI = [0.00, 0.21], *p* = 0.042; Fig. 5, Table S8).

## Discussion

In Study 1, we set out to investigate the extent to which embodied dance experience acquired through training and differences in individuals’ performance abilities impact behavioural and cortical perceptions of action aesthetics. In Study 2, we explored how individuals’ previous engagement with arts and sports shape aesthetic judgements of action. We found that the relationships between different aesthetic judgements (positive for familiarity and reproducibility, as well as familiarity and enjoyment, negative for reproducibility and enjoyment) are consistent with previous reports^2,3,11,17^, and that one’s own embodied and cumulative experiences are, in fact, likely to influence perceptions of full-body movements.

### Embodied experience, but not performance ability, predicts aesthetic judgements

In Study 1, we found that learning a short choreography resulted in higher ratings of enjoyment, familiarity, and reproducibility of body movements overall, but more so for movements belonging to the learned choreography. This is consistent with previous findings demonstrating that physical practice and embodied experience of specific movements, as opposed to merely watching said movements, enhance aesthetic appreciation of the movements^26,30^. Following from previous work by Kirsch et al.^2^, we expected that participants’ ability to perform the choreography might predict their aesthetic perceptions of learned movements. We did not observe this in Study 1, rather we observed no relationship between participants’ performance ability and their ratings for enjoyment, familiarity, or reproducibility (Fig. 1C). A possible explanation for this discrepancy is that our ability measure, i.e., ratings between 1 and 10 by two experienced dancers, may not have been sufficiently sensitive to capture subtle differences in embodied experience. Future research could implement a more sensitive measure, such as a computer-vision approach comparing the similarity of the movements performed by participants and by the original dancer in the stimulus videos^31,32^.

### Dance performance predicts cortical responses to known movements

Our exploratory analyses, however, did show a relationship between cortical activation in bilateral IFG and right MTG and performance ability (Fig. 3B, Figure S3B). Cortical activity, as measured by HbO, while viewing known movement sequences was less negative (closer to zero). The negative estimates for both HbO and HbR in these ROIs for this time-period (the first 6 s from video-onset) may reflect the first stages (i.e., anticipation, visual perception and early, automatic formation of aesthetic judgement) of the complex processing required across each trial (i.e., visual perception, formation, and delivery of explicit judgement)^33,34^. Note: Further discussion of inverted haemodynamic responses (also called negative BOLD responses) can be found in Supplementary Materials, pages 2–3. Following the assumption that the first 6 s reflect early, automatic aesthetic processing, we must ponder why individuals who were less able to perform the choreography showed stronger inverted haemodynamic responses (i.e., HbO more negative that HbR) only during the early stages, and not the later stages of the time course of cortical activation.

With respect to the early stages of aesthetic action processing, greater experience with the movements may reduce the degree of simulation needed for the visuo-motor system to identify the sequence as known. We draw support for this, tentatively presented, interpretation from electroencephalography (EEG). In EEG recordings, motor simulation seems to evoke increased alpha power (8–13 Hz) and decreased beta (13–26 Hz) power^35^. Alpha power, associated with top-down attention^36^, correlates negatively with haemodynamic activation in frontal regions including IFG^37^. Beta power is characteristic of executed, observed, or imagined body movements^38,39^. Beta power correlates positively with haemodynamic activity in the superior temporal gyrus^37^, which likely contributes to the signal that we measured over the MTG using fNIRS, given fNIRS’ spatial resolution of 2–3 cm^40^. Perhaps, negative haemodynamic responses in the IFG could plausibly be associated with increased alpha power, and negative haemodynamic responses in MTG could plausibly be associated with decreased beta power–consistent with power profile of motor simulation. We thus speculate the degree of motor simulation while watching known movement sequences may be greater for poor performers than good performers, as indicated by the positive correlation between performance and HbO in bilateral IFG (left trending only) and bilateral MTG. This speculation is in line with previous work demonstrating that expert dancers, relative to non-dancers, show reduced alpha power and increased beta power when viewing dance sequences^41^. Bolstering the plausibility of this interpretation further, unknown movement sequences evoked negative haemodynamic responses in the IFG and MTG that did not correlate with performance, suggesting that all participants engaged in substantial motor simulation while viewing unknown movement sequences. In other words, relative to poor performers, participants with better performance scores may engage in less motor simulation while viewing known movements and the same amount of motor simulation while viewing unknown movements.

Across the whole viewing and rating time window (13-s boxcar), we observed no relationship between performance ability and cortical activation in any ROI, suggesting that individual differences in the level of embodiment, as evaluated by outside observers (i.e., performance ability), may be more influential while encoding movements rather than while forming aesthetic judgements. This finding is, however, at odds with work by Kirsch & Cross^8^, who demonstrated that enjoyment of full-body movements is positively correlated with activity in the angular gyrus using fMRI. The placement of optodes over the IPL in the present work also covers the angular gyrus^40^. It is possible that the cortical activation from the angular gyrus was too focal to result in substantial activation in the IPL ROI. A second possibility is that, as mentioned above, our measure of performance ability could benefit from added sensitivity. Finally, the training manipulation in Kirsch & Cross’ study involved multiple days of training, i.e., much more training than the approximately 20 min provided in the present study.

We are open to the possibility that our measure of performance ability (ratings by two experienced dancers) may not be the most sensitive method of indexing evidence of embodiment. Future studies stand to benefit from implementing more objective measures of performance. For example, OpenPose^42^ or other pose-detection software could be used to assess the similarity between participants’ and original dancer’s body positions over time^31^, which would yield a more sensitive and objective measure of performance ability. Further, pose detection software could be used to obtain an objective measure of complexity, such as entropy^11,43,44^ from each movement sequence, as complexity is known to influence aesthetic judgements. This measure of movement complexity may be included as a regressor in statistical analyses^10,11,14^. Such an approach to quantifying performance ability could shed more fine-grained insight on the relationship between performance ability and cortical activation in bilateral IFG and right MTG (Fig. 3B, Figure S3B).

### Individual differences in engagement with the arts influence ratings of familiarity and reproducibility, but not enjoyment

Our data from Study 2, examining individual differences in experience with and enjoyment of the arts, suggested that engagement with certain types of arts or sports may shape evaluations of body movements. Ratings of familiarity were correlated with experience painting and dancing (in informal settings, as individuals with dance training were excluded from this study), as well as enjoyment of theatre and social media dance. The link between painting and dancing experience and ratings of familiarity could stem from the detailed observations of body postures and movements required to transfer poses to canvas or recreate the movements oneself^14^, especially when kinematic information is involved^45^. With respect to the association between familiarity ratings and enjoyment of theatre and dance on social media, we propose that both art types incorporate dramatic full-body movements which may have resembled those shown in the present study^46^. These findings highlight how cumulative exposure to body movements through the lens of certain art types may bolster fluency, as observed in participants’ ratings of familiarity.

Ratings of reproducibility, a self-report proxy for embodiment^47^, were positively associated with reported enjoyment of sports, music, and dance on social media. We propose that participants’ enjoyment of sports is likely linked to time spent watching sporting events (although this was not quantified in the present work), and that this cumulative exposure may result in a stronger belief that the observed movements are not beyond the participant’s physical capacities. Interestingly, we did not find ratings of reproducibility to be correlated with experience with sports. Evidence from our research group suggests that individual differences in the attention that one pays to one’s bodily functions and the confidence that one has in one’s body’s physical capacities are correlated with ratings of reproducibility when observing snippets of a mirror game^43^. A possible link between sports enjoyment or experience and body confidence and action intention should be considered in future work^23,48^. As for the relationship between enjoyment of music and ratings of reproducibility, we suggest that perhaps participants who enjoy music more are better attuned to beats and rhythms, even when no music or auditory beat is present^49^. Such participants may have extracted visual rhythmic information from the choreographies or experienced a form of entrainment. The choreographies were performed at a steady rhythm, with no variations in timing, perhaps simplifying motor simulation for those with developed rhythm perception^50^, especially given the tight relationship between rhythm and motor perception^51,52^. Considered together, these findings offer insight into which art forms, including sports more generally, are most likely to offer avenues to embodiment of a wider variety of movements.

We found that enjoyment of dance on social media (e.g., TikTok dances) was positively associated with familiarity ratings, while experience with social media dance was correlated with reproducibility ratings. Increased exposure to dance on social media may heighten familiarity, or fluency, with body movements and, for certain individuals who try or master trending choreographies, increase the variety of movements they embody. Interestingly, neither experience nor enjoyment of dance on social media influenced enjoyment of the dance movements observed in Study 2. One limitation of Study 2 is that we did not query how often participants practiced or otherwise physically engaged with dances from social media. This information could deepen our understanding of the influence of social media dance on learning, understanding, and subsequently performing full-body movements in social and communicative settings. Further, it is important to consider the effect of social media engagement on young people’s development, given that emerging research reports engagement with social media to be both positive (e.g., enhanced multicultural experience, cultural intelligence, individual creativity)^53^ and negative (i.e., disordered body image, low self-esteem)^54^ for young people’s development. An opportunity clearly exists for further research to enhance our understanding of the extent to which engagement in art-based trends (i.e., dance, as opposed to dangerous behaviours) might impact its consumers.

We were surprised that no forms of experience with or enjoyment of arts or sports queried in Study 2 were associated with enjoyment of movement sequences. We expected participants with higher levels of experience with and enjoyment of arts and sports to report greater enjoyment, based on potentially greater fluency of processing dynamic body movements. While we might expect heightened fluency of processing, this may not translate to enjoyment of full-body movements in a dance context. In other words, enjoyment of dynamic movements may be more strongly influenced by dance-specific expertise than experience with the arts in general.

Moreover, the role of individual experiences with arts and sports in aesthetic perceptions aligns with the subsystems of the Aesthetic Triad model: more frequent engagement with or physical practice likely strengthens sensory–motor, emotion–valuation, as well as meaning–knowledge related processing^4^. However, the exact links between the model and individual’s unique profiles remain to be systematically investigated. Future work should further consider socio-cultural aspects beyond experience with sports and arts, such as religious beliefs, socio-economic status, and social connectedness.

## Conclusions

In this pair of studies, we examined how embodied experience and performance ability shape aesthetic action perception and cortical activation evoked by known and unknown movements (Study 1). We then explored the influence of individual differences in arts and sports engagement on aesthetic perceptions of movement (Study 2). Our findings from Study 1 suggest that gaining embodied experience through dance training increases ratings of enjoyment, familiarity, and reproducibility of full-body movements, while also reducing the strength of inverted haemodynamic responses recorded over the bilateral IFG and right MTG during observation of known movements. These findings support the theoretical relevance of embodiment in explaining aesthetic appreciation of body movements. Study 2 demonstrated that engagement with certain art forms or sports can increase ratings of familiarity (i.e., processing fluency) and reproducibility (i.e., perceived embodiment), but not enjoyment, of movements. This suggests that arts experience may enhance embodiment of movements quite generally, but not sufficiently to have the same impact as acutely embodied experience on aesthetic appreciation. Considering these studies together, we propose the influence of embodiment on aesthetic perceptions may be greater than the influence of individual differences in experience with other arts and movement-based activities, as measured here. Nonetheless, our explicit consideration of how an individual’s experiences can shape their perception of body movements offers the field of action aesthetics new directions embedded in social contexts.

## Materials and methods

### Study 1

**Fig. 6:**
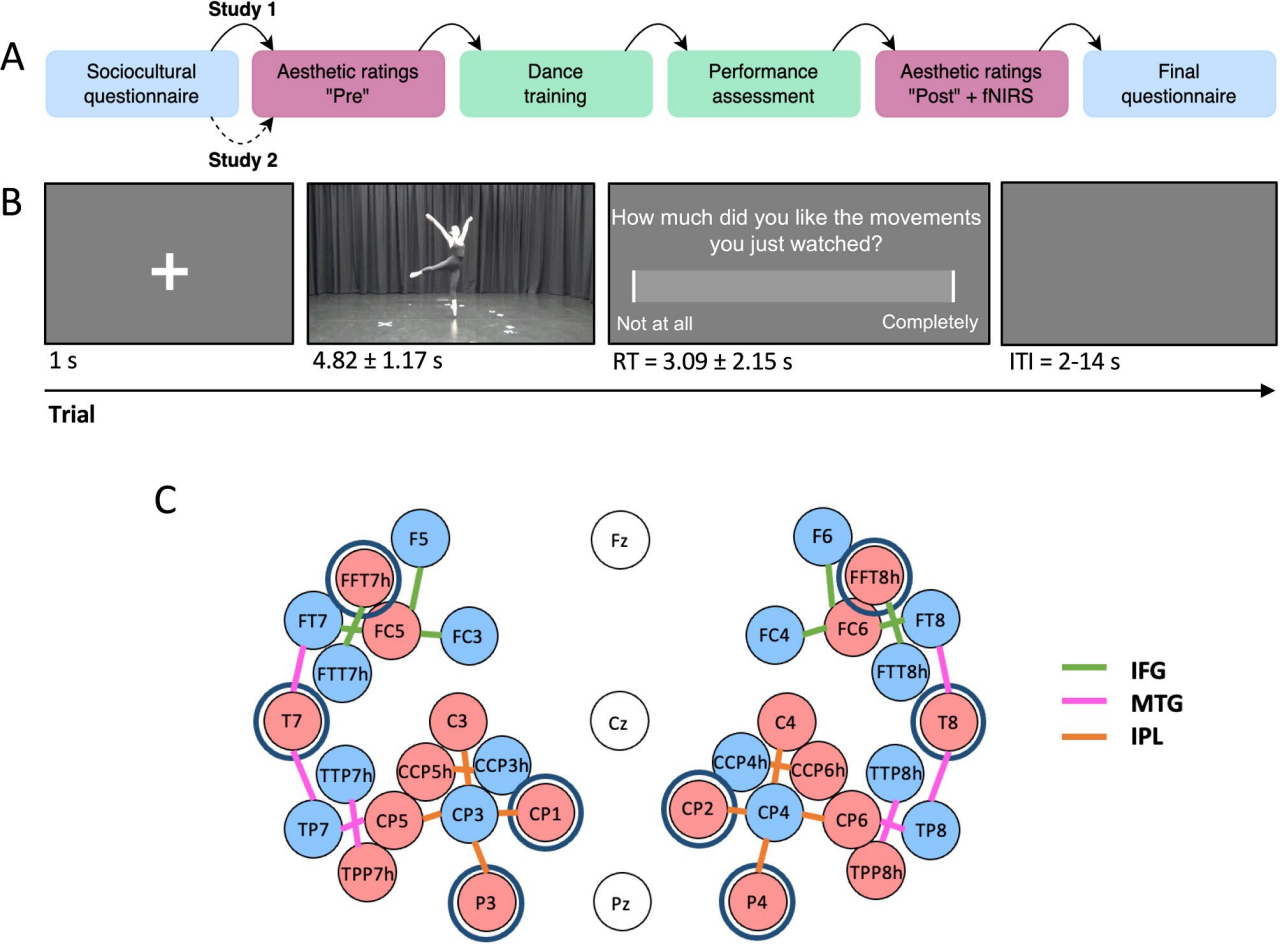
(**A**) Procedure of Studies 1 and 2. Study 1 consisted of all components, and Study 2 only of the first two components. (**B**) Schematic of one trial in the aesthetic rating task, consisting of 48 trials (3 × 16 videos). The intertrial-interval (ITI) indicates the jittered onset of trials during fNIRS recordings. (**C**) fNIRS montage of sources (red circles) and detectors (light blue circles), and short-detector channels (navy circles) using International 10/10 positions. Lines connecting sources and detectors show channels belonging to each ROI: IFG (green), IPL (orange), and MTG (pink).

Study 1 consisted of a sociocultural questionnaire, a “Pre” aesthetic rating task, a dance training session, a “Post” aesthetic rating task (same as “Pre”) with concurrent fNIRS measurement, and a final questionnaire (Fig. 6A).

#### Participants

Forty-one participants (21.59 ± 4.72 years; 26 female/14 male/1 prefered not to respond; 38 right-handed/3 left-handed) were recruited from a pool of undergraduate psychology students at Macquarie University and from the wider community in Sydney, Australia. All participants were 18–40 years old, had no dance experience beyond basic familiarity (e.g., took ballet classes as a child), had no history of neurological or psychiatric condition (e.g., attention-deficit/hyperactivity disorder, depression, autism, schizophrenia, epilepsy) or of head injury (e.g., concussion), did not take psycho-pharmaceutical medication (e.g., SSRIs, Ritalin), and did not consume THC or alcohol in the 24 h before the study. Ethical approval for this study was obtained from the Macquarie University Human Research Ethics Committee (Ref: 520221199641492). All participants provided written informed consent and this research was undertaken in accordance with the Declaration of Helsinki. Participants received AUD $40 or course credits for their involvement.

As per our preregistered aim to embrace sociocultural diversity in our sampling of participants, this sample spanned a wide range of cultural backgrounds, including those with darker and thicker hair^55^. This had a substantial impact on signal quality in a subset of participants. Visual inspection of scalp-coupling indices^56^ per participant revealed this to be most severe in three participants, and mild in another seven (Figure S1 in Supplementary Materials), who we excluded from the group-level analyses of cortical activation, yielding 31 participants with clean signals. All 41 participants were included in analyses of behavioural data in Study 2.

#### Sociocultural and final questionnaires

First, participants completed a questionnaire administered via REDCap® that queried demographics (e.g., age, gender), cultural background (e.g., ethnicity, religion), as well as previous engagement with the arts and sports, and dance efficacy. Dance efficacy refers to one’s self-assessed reflection of individual factors likely to influence dance participation and other wellbeing outcomes^22^. The final questionnaire included questions designed to probe participants’ feelings toward dance and other elements of the study after having gained embodied dance experience, which are not analysed here. Full questionnaires available on OSF (https://osf.io/cndfg/).

#### Aesthetic rating task

Next, participants viewed videos of sequences of full-body movements, or choreographies. In this “Pre” assessment, all videos are *unknown* to participants. Subsequently participants completed a dance training ("Dance training and assessment of performance ability"), in which they learned movements from half of the videos. Their learning was assessed through a video-recorded performance, then they completed the same aesthetic rating task again. As a result of the training, half of the movement sequences were now *known* or learned, while half remained *unknown* in the “Post” rating task.

#### Stimuli

Two 30-s choreographies of simple, original dance movements served as the basis for the aesthetic assessments and for the dance training phase of the study. For the aesthetic assessments, each of the two 30-s choreographies was divided into 8 distinct movement sequences (total n = 16). These were recorded as short video clips (ranging from 3 to 7 s in length, mean = 4.82 s, SD = 1.17 s) and are available on OSF (https://osf.io/jfv43/). All videos were recorded with a Sony HDR-PJ410 video camera at the MARCS Institute of Western Sydney University.

#### Presentation

Participants viewed videos of movement sequences then answered rating questions probing aesthetic perceptions. We measured subjective enjoyment using the question “How much did you like the movements you just watched?”. To measure visual expertise (i.e., fluency of processing), we asked “How familiar did you find the movements you just watched?”. We measured perceived motor expertise (i.e., embodiment) using “How reproducible did you find the movements you just watched?”. Participants responded using a computer mouse to click on a sliding scale that showed no numbers and ranged from “Not at all” to “Completely” (Fig. 6B). “Not at all” was associated with a value of zero, and “Completely” with 100.

Each trial consisted of a fixation cross (1 s), then a video showing a sequence of movements (~ 5 s), following by a single rating question (i.e., liking, familiarity, reproducibility) with a slider (Fig. 6B). Each video was presented three times to ensure participants completed ratings for all three questions. Both videos and questions were presented in a randomised order on a desktop computer via PsychoPy (version 2021.2.3)^57^. In the “Pre” task, the intertrial-interval (ITI) was 2 s from the participant’s selection of a rating response. In the “Post” task, where fNIRS was used to measure changes in cortical oxygenation, we used a jittered ITI (8–14 s) from the participant’s response selection. We included one practice trial using a nature video (rated for enjoyment) before the first trial to familiarise participants with the procedure.

#### Dance training and assessment of performance ability

Participants were randomly assigned to learn one of two choreographies. In this way, we counterbalanced the known and unknown videos across our sample. Participants followed a training video that included multiple repetitions of the steps broken down individually and slowly at first, and then all together and faster as the video progressed. Participants were instructed to follow along with the dancer in the video and physically repeat the movements each time they were shown on the screen. Dance training videos are available on OSF (https://osf.io/jfv43/). Creation, performance, and instruction of all choreography was done by CEC, a trained dancer. Both pieces of choreography were determined to be of approximately the same level of difficulty by the research team.

Following the dance training, the researcher video-recorded participants performing the learned choreography. Participants’ performance ability, i.e., execution of the learned choreography, was scored on a scale from 1 to 10 (1 = “poor”, 10 = “perfect”) by two experienced dancers (CEC and Andrea Orlandi). Scores given by CEC and AO were averaged per participant to calculate each participant’s performance ability score.

#### fNIRS equipment and optode positioning

While participants viewed the sequences of movements a second time, after the dance training, we measured changes in cortical oxygenation, a proxy for cortical activation, using fNIRS. fNIRS measurements were recorded with a NIRScout (NIRx Medical Technologies LLC) with 24 LED light sources and 32 avalanche photodiode detectors. Sources emitted near-infrared light at wavelengths of 760 and 850 nm with a sampling rate of 3.47 Hz. Optodes (i.e., sources and detectors) were attached onto mesh caps (Easycap GmbH) using International 10/10 positions as a guide. Grommets and spacers were used to maintain a maximum of 30 mm of separation between all source-detector pairs (NIRx Medical Technologies LLC). This spacing was employed to ensure the recorded signal comes from the correct depth below the scalp (1.5 cm) and to maximise the signal-to-noise ratio^58^.

Changes in cortical oxygenation, a proxy for cortical activation, were recorded from bilateral IFG, IPL, and MTG using a montage of 18 sources, 16 detectors, and 8 short-channel detectors (Fig. 6C). We used the AAL2 atlas in the fOLD toolbox^40,59,60^ to select optode positions. The montage consisted of 26 long channels (source-detector pairs with ~ 30 mm of separation) and 8 short channels (source-detector pairs with 8 mm of separation). Short detectors were distributed across ROIs (1 in each IFG, 1 in each MTG, and 2 in each IPL) to account for heterogeneity of haemodynamic signals across the scalp^61^.

#### Procedure

In study 1, participants completed the following activities: A sociocultural questionnaire, a “Pre” aesthetic rating task, a dance training session, a “Post” aesthetic rating task (same stimuli as “Pre” with longer ITIs) with concurrent fNIRS measurement, and a final questionnaire (Fig. 6A). The experiment lasted approximately 2 h.

#### Data analysis

Statistical analyses probed the extent to which participants’ neural and behavioural responses, to the movement sequences presented in the videos, changed as a function of the embodied expertise acquired through dance training. Cortical data recorded using fNIRS required individual- and group-level analyses, whereas behavioural data only required group-level analyses. All analyses were preregistered, and data are available on OSF (https://osf.io/cndfg/).

##### Individual-level analyses–cortical activity

Analyses of haemodynamic response amplitude^62^ were performed using MNE (version 1.2.2)^63^, MNE-NIRS (version 0.0.4)^64^, and NiLearn (version 0.9.2)^65^. We first inspected the signal quality using a scalp-coupling index for frequencies between 0.7–1.35 Hz (Figure S1 of Supplementary Materials)^56^. Next, we generated waveforms for visual inspection (procedure detailed in Supplementary Materials), which led us to fit two separate GLMs (discussed in "Unique contributions of viewing and evaluating movement to cortical activation"), with 6-s and 13-s boxcar functions respectively. The calculation supporting these boxcar lengths are: For 6 s = mean duration of video viewing (1 s fixation cross + 4.82 s [mean video length], rounded up from 5.82 s to 6 s). For 12.93 s = mean time to preparation and selection of aesthetic rating (1 s fixation cross + 6.69 s [mean question onset] + 3.09 s [mean response time] + 2.15 s [1 standard deviation of response time], rounded up to 13 s).

Prior to quantifying the amplitude of evoked haemodynamic responses measured using fNIRS per participant, we took the following steps: First, raw absolute intensity values recorded by the NIRScoutX were converted to optical density, which was further converted to concentrations of oxygenated and deoxygenated haemoglobin (HbO and HbR, respectively) using the Modified Beer-Lambert Law^66,67^. We used a partial pathlength factor of 0.1, which accounts for both differential pathlength factor (DPF) and partial volume correction (PVC), where (DPF = 6)/(PVC = 60) is equal to 0.1^68,69^. Next, channels < 20 mm or > 40 mm were excluded, meaning that we fit each GLM using channels (20–30 mm) expected to measure from ~ 1.5 cm below the scalp^58^. The design matrix for each GLM was generated by convolving the selected boxcar function (6 or 13 s) with the canonical haemodynamic response function^70^. All principal components of short-detector channels were also included in the GLM to account for extracerebral and physiological signal components, and drift orders accounting for signal components up to 0.01 Hz were included as regression factors^62^. Moreover, to account for the correlated nature of the fNIRS signal components, the GLM was performed with a lag-1 autoregressive noise model^62^. Finally, estimates extracted from the GLM were averaged for each ROI in the brain, weighted by the standard error. These were entered into separate group-level analyses per boxcar function (i.e., 6s and 13s).

##### Group-level analyses–Behavioural responses and cortical activity

We fit linear mixed-effects models using R (version 4.2.1)^71^ in the RStudio IDE (version 2022.07.2)^72^. Models were constructed in a step-wise fashion using R package lme4 (version 1.1–30)^73^. We added terms one-by-one, starting with random effects and then adding fixed effects incrementally^74,75^. The maximal number of terms as allowed by model comparison (using Akaike’s Information Criterion, AIC) was retained. While modelling group-level cortical activation, we suppressed the intercept to compare each predictor term against zero, i.e., to determine whether the predicted amplitude of a haemodynamic response is significantly different from zero for a given ROI and condition^68^. For analyses of both behavioural and cortical responses, we used the emmeans R package (version 1.8.4–1)^76^ to contrast relevant parameter estimates and the false discovery rate procedure^77^ to correct contrasts for multiple comparisons.

### Study 2

Study 2 comprised the sociocultural questionnaire and the “Pre” aesthetic rating task described above for Study 1 (Fig. 6).

#### Participants

One hundred participants (20.71 ± 3.76 years; 79 female, 17 male, 3 other, 1 prefered not to say; 84 right-handed, 13 left-handed, 3 ambidextrous) were recruited from a pool of undergraduate psychology students at Macquarie University and from the wider community in Sydney, Australia. All participants were 18–40 years old and had no dance experience beyond basic familiarity (e.g., took ballet classes as a child). Ethical approval for this study was obtained from the Macquarie University Human Research Ethics Committee (Ref: 520221199641492). All participants provided written informed consent and this research was undertaken in accordance with the Declaration of Helsinki. Participants received AUD $10 or course credits for their involvement.

#### Procedure

After providing written informed consent to take part in the study, participants completed the sociocultural questionnaire, described in "Sociocultural and final questionnaires", with additional measures of sports enjoyment, dance enjoyment, and social media dance enjoyment. Next participants completed the “Pre” aesthetic rating task ("Aesthetic rating task"). The experiment lasted approximately 30 min.

#### Data analysis

Data related to previous arts and sports experience from Study 1 and Study 2 were analysed together, using data from all 141 participants. Sports enjoyment, dance enjoyment, and social media dance enjoyment were added to the sociocultural questionnaire for Study 2, meaning that these variables were not available for participants from Study 1 (n = 41). To address all measures (including these 3 new ones) in the same models, we imputed missing values using the mean value per measure (i.e., the average across 100 participants in Study 2 for each sports enjoyment, dance enjoyment, and social media dance enjoyment). This allowed research questions probing the influence of these new measures alongside measures collected in both studies to be addressed in the same models. Our preregistered analyses and all data are available under the Open Science Framework (https://osf.io/vfsn2/).

## Supplementary Information

Supplementary Information.

## Data Availability

The stimuli, datasets, and code used in this work can be found on OSF: Stimuli: “Aesthetic Processing of Dynamic Movement: Dance Video Stimuli”, https://osf.io/jfv43/; Study 1: “Aesthetic Processing of Dynamic Movement: Evidence from Brain and Behaviour”, https://osf.io/cndfg/; Study 2: “Aesthetic Preferences of Human Movement: Does Experience with the Arts Matter?”, https://osf.io/vfsn2/.

## References

[1] Blacking, J. Movement and meaning: Dance in social anthropological perspective. *Dance Res.*** 1**, 89–99 (1983).

[2] Kirsch, L. P., Dawson, K. & Cross, E. S. Dance experience sculpts aesthetic perception and related brain circuits. *Ann. N. Y. Acad. Sci.*** 1337**, 130–139 (2015). 25773627 10.1111/nyas.12634 PMC4402020

[3] Orgs, G., Caspersen, D. & Haggard, P. You move, I watch, it matters: Aesthetic communication in dance. In *Shared Representations* 1st edn (eds Obhi, S. S. et al.) 627–653 (Cambridge University Press, 2016).

[4] Vartanian, O. & Chatterjee, A. The aesthetic triad. In *Brain, Beauty, and Art: Essays Bringing Neuroaesthetics into Focus* 1st edn (eds Chatterjee, A. & Cardilo, E.) 27–30 (Oxford University Press, 2021).

[5] Darda, K. M. & Cross, E. S. The role of expertise and culture in visual art appreciation. *Sci. Rep.*** 12**, 10666 (2022). 35739137 10.1038/s41598-022-14128-7 PMC9219380

[6] Cross, E. S. & Orlandi, A. The aesthetics of action and movement. In *The Oxford Handbook of Empirical Aesthetics* 1st edn (eds Nadal, M. & Vartanian, O.) 605–622 (Oxford University Press, 2020).

[7] Kirsch, L. P., Urgesi, C. & Cross, E. S. Shaping and reshaping the aesthetic brain: Emerging perspectives on the neurobiology of embodied aesthetics. *Neurosci. Biobehav. Rev.*** 62**, 56–68 (2016). 26698020 10.1016/j.neubiorev.2015.12.005

[8] Kirsch, L. P. & Cross, E. S. The influence of sensorimotor experience on the aesthetic evaluation of dance across the life span. In *Progress in Brain Research*, Vol. 237 (eds Christensen J. F. & Gomila, A.) 291–316 (Elsevier, 2018). 10.1016/bs.pbr.2018.03.012 29779740

[9] Wang, Z. Evaluation of Creativity in Contemporary Dance in Terms of Audience Perception. *Creat. Res. J.*** 36**, 234 (2022).

[10] Cross, E. S., Kirsch, L., Ticini, L. F. & Schütz-Bosbach, S. The impact of aesthetic evaluation and physical ability on dance perception. *Front. Hum. Neurosci * (2011). 21960969 10.3389/fnhum.2011.00102 PMC3177045

[11] Orlandi, A., Cross, E. S. & Orgs, G. Timing is everything: Dance aesthetics depend on the complexity of movement kinematics. *Cognition*** 205**, 104446 (2020). 32932073 10.1016/j.cognition.2020.104446

[12] Reber, R., Schwarz, N. & Winkielman, P. Processing fluency and aesthetic pleasure: Is beauty in the perceiver’s processing experience?. *Personal. Soc. Psychol. Rev.*** 8**, 364–382 (2004). 10.1207/s15327957pspr0804_3 15582859

[13] Zeki, S. *Inner Vision: An Exploration of Art and the Brain* (Oxford University Press, 1999).

[14] Orgs, G., Hagura, N. & Haggard, P. Learning to like it: aesthetic perception of choreographic patterns. *Cogn. Process.*** 13**, S28–S29 (2013). 10.1016/j.concog.2013.03.010 23624142

[15] Berg, S. C. Le Sacre Du Printemps: Seven Productions from Nijinsky to Martha Graham. https://dokumen.pub/le-sacre-du-printemps-seven-productions-from-nijinsky-to-martha-graham-0835718425-9780835718424.html (1988).

[16] Carbon, C. C. The cycle of preference: Long-term dynamics of aesthetic appreciation. *Acta Psychol.*** 134**, 233–244 (2010). 10.1016/j.actpsy.2010.02.004 20236624

[17] Vinken, P. M. & Heinen, T. How does the amount of movement and observer expertise shape the perception of motion aesthetics in dance?. *Hum. Mov.*** 23**, 46–55 (2022).

[18] Department for Culture, Media and Sport. Taking Part focus on: Cross-sector participation [Statistical Release] (2016). https://www.gov.uk/government/statistics/taking-part-april-2016-focus-on-reports.

[19] Belk, R. W., Semenik, R. J. & Andreasen, A. R. Co-patronage patterns in arts-related leisure activities. In *SV-Consumer Behavior*, Vol. SV-04 (eds Hirschman, E. C. & Holbrook, M. B.) 95–100 (Association for Consumer Research, 1981). https://www.acrwebsite.org/volumes/12236/volumes/sv04/SV-04/full.

[20] Monroy, E., Imada, T., Sagiv, N. & Orgs, G. Dance Across Cultures: Joint Action Aesthetics in Japan and the UK. *Empir. Stud. Arts*** 40**, 209–227 (2022).

[21] Rose, D., Müllensiefen, D., Lovatt, P. & Orgs, G. The Goldsmiths Dance Sophistication Index (Gold-DSI): A psychometric tool to assess individual differences in dance experience. *Psychol. Aesthet. Creat. Arts* (2020).

[22] Waugh, M. *So you think you can dance? Investigating perceived dance efficacy and dance program participation in older adults* (Western Sydney University, 2022).

[23] Sevdalis, V. & Raab, M. Individual differences in athletes’ perception of expressive body movements. *Psychol. Sport Exerc.*** 24**, 111–117 (2016).

[24] Cross, E. S., Kraemer, D. J. M., Hamilton, A. F. d. C., Kelley, W. M. & Grafton, S. T. Sensitivity of the Action Observation Network to Physical and Observational Learning. *Cereb. Cortex*** 19**, 315–326 (2009). 10.1093/cercor/bhn083 PMC2638791 18515297

[25] Ono, Y. et al. Motor learning and modulation of prefrontal cortex: an fNIRS assessment. *J. Neural Eng.*** 12**, 066004 (2015). 26401727 10.1088/1741-2560/12/6/066004

[26] Sumanapala, D. K., Fish, L. A., Jones, A. L. & Cross, E. S. Have I grooved to this before? Discriminating practised and observed actions in a novel context. *Acta Psychol. (Amst.)*** 175**, 42–49 (2017). 28284106 10.1016/j.actpsy.2017.02.008

[27] Freedberg, D. & Gallese, V. Motion, emotion and empathy in esthetic experience. *Trends Cogn. Sci.*** 11**, 197–203 (2007). 17347026 10.1016/j.tics.2007.02.003

[28] Pinti, P. et al. The present and future use of functional near-infrared spectroscopy (fNIRS) for cognitive neuroscience. *Ann. N. Y. Acad. Sci.*** 1464**, 5–29 (2020). 30085354 10.1111/nyas.13948 PMC6367070

[29] Yokota, H., Kamijo, K., Mizuguchi, N., Kubo, H. & Nakata, H. Motor imagery and action observation of whole-body movements for experienced motor repertoire: an fNIRS study. *J. Sports Med. Phys. Fitness*** 12**, 107–117 (2023).

[30] Calvo-Merino, B., Grèzes, J., Glaser, D. E., Passingham, R. E. & Haggard, P. Seeing or doing? Influence of visual and motor familiarity in action observation. *Curr. Biol.*** 16**, 1905–1910 (2006). 17027486 10.1016/j.cub.2006.07.065

[31] Moffat, R., Caruana, N. & Cross, E. S. Inhibiting responses under the watch of a recently synchronized peer increases self-monitoring: evidence from functional near-infrared spectroscopy. *Open Biol. *(2024). 38378138 10.1098/rsob.230382 PMC10878812

[32] Broadwell, P. & Tangherlini, T. R. Comparative K-Pop Choreography Analysis through Deep-Learning Pose Estimation across a Large Video Corpus. *Digit. Hum. Q.*** 15**, 1–25 (2021).

[33] Chatterjee, A., Thomas, A., Smith, S. E. & Aguirre, G. K. The neural response to facial attractiveness. *Neuropsychology*** 23**, 135–143 (2009). 19254086 10.1037/a0014430

[34] Cela-Conde, C. J. et al. Dynamics of brain networks in the aesthetic appreciation. *Proc. Natl. Acad. Sci.*** 110**, 10454–10461 (2013). 23754437 10.1073/pnas.1302855110 PMC3690613

[35] Siqi-Liu, A., Harris, A. M., Atkinson, A. P. & Reed, C. L. Dissociable processing of emotional and neutral body movements revealed by μ-alpha and beta rhythms. *Soc. Cogn. Affect. Neurosci.*** 13**, 1269–1279 (2018). 30351422 10.1093/scan/nsy094 PMC6277737

[36] Hobson, H. M. & Bishop, D. V. M. Mu suppression – A good measure of the human mirror neuron system?. *Cortex*** 82**, 290–310 (2016). 27180217 10.1016/j.cortex.2016.03.019 PMC4981432

[37] Michels, L. et al. Simultaneous EEG-fMRI during a Working Memory Task: Modulations in Low and High Frequency Bands. *PLoS one *(2010). 20421978 10.1371/journal.pone.0010298 PMC2858659

[38] Babiloni, C. et al. Human Cortical Electroencephalography (EEG) Rhythms during the Observation of Simple Aimless Movements: A High-Resolution EEG Study. *NeuroImage*** 17**, 559–572 (2002). 12377134

[39] McFarland, D. J., Miner, L. A., Vaughan, T. M. & Wolpaw, J. R. Mu and Beta Rhythm Topographies During Motor Imagery and Actual Movements. *Brain Topogr.*** 12**, 177–186 (2000). 10791681 10.1023/a:1023437823106

[40] Zimeo Morais, G. A., Balardin, J. B. & Sato, J. R. fNIRS Optodes’ Location Decider (fOLD): a toolbox for probe arrangement guided by brain regions-of-interest. *Sci. Rep.*** 8**, 3341 (2018). 29463928 10.1038/s41598-018-21716-z PMC5820343

[41] Di Nota, P. M., Chartrand, J. M., Levkov, G. R., Montefusco-Siegmund, R. & DeSouza, J. F. X. Experience-dependent modulation of alpha and beta during action observation and motor imagery. *BMC Neurosci.*** 18**, 28 (2017). 28264664 10.1186/s12868-017-0349-0 PMC5340035

[42] Cao, Z., Hidalgo, G., Simon, T., Wei, S.-E. & Sheikh, Y. OpenPose: Realtime Multi-Person 2D Pose Estimation using Part Affinity Fields. Preprint at arXiv:1812.08008 (2019). 10.1109/TPAMI.2019.2929257 31331883

[43] Moffat, R. & Cross, E. S. Evaluations of dyadic synchrony: observers’ traits influence estimation and enjoyment of synchrony in mirror-game movements. *Sci. Rep.*** 14**, 2904 (2024). 38316911 10.1038/s41598-024-53191-0 PMC10844651

[44] Zhou, J. *et al.* Skeleton-based Human Keypoints Detection and Action Similarity Assessment for Fitness Assistance. *2021 IEEE 6th Int. Conf. Signal Image Process. *(2021).

[45] Gray, J. T., Neisser, U., Shapiro, B. A. & Kouns, S. Observational Learning of Ballet Sequences: The Role of Kinematic Information. *Ecol. Psychol.*** 3**, 121–134 (1991).

[46] Prousali, E. A Neuroaesthetic approach to Performance Perception. 17/2, (2022).

[47] Cross, E. S., Hamilton, A. F. & Grafton, S. T. Building a motor simulation de novo: Observation of dance by dancers. *NeuroImage*** 31**, 1257–1267 (2006). 16530429 10.1016/j.neuroimage.2006.01.033 PMC1821082

[48] Sevdalis, V. & Keller, P. E. Captured by motion: dance, action understanding, and social cognition. *Brain Cogn.*** 77**, 231–236 (2011). 21880410 10.1016/j.bandc.2011.08.005

[49] Trost, W. J., Labbé, C. & Grandjean, D. Rhythmic entrainment as a musical affect induction mechanism. *Neuropsychologia*** 96**, 96–110 (2017). 28069444 10.1016/j.neuropsychologia.2017.01.004

[50] Sánchez, C. V. *Rhythm. Int. Lex. Aesthet. *(2022).

[51] Ross, J. M., Iversen, J. R. & Balasubramaniam, R. Motor simulation theories of musical beat perception. *Neurocase*** 22**, 558–565 (2016). 27726485 10.1080/13554794.2016.1242756

[52] Karpati, F. J., Giacosa, C., Foster, N. E. V., Penhune, V. B. & Hyde, K. L. Dance and music share gray matter structural correlates. *Brain Res.*** 1657**, 62–73 (2017). 27923638 10.1016/j.brainres.2016.11.029

[53] Hu, S., Gu, J., Liu, H. & Huang, Q. The moderating role of social media usage in the relationship among multicultural experiences, cultural intelligence, and individual creativity. *Inf. Technol. People*** 30**, 265–281 (2017).

[54] Pruccoli, J., De Rosa, M., Chiasso, L., Perrone, A. & Parmeggiani, A. The use of TikTok among children and adolescents with Eating Disorders: experience in a third-level public Italian center during the SARS-CoV-2 pandemic. *Ital. J. Pediatr.*** 48**, 138 (2022). 35907912 10.1186/s13052-022-01308-4 PMC9338669

[55] Kwasa, J. et al. Demographic reporting and phenotypic exclusion in fNIRS. *Front. Neurosci. *(2023). 37229429 10.3389/fnins.2023.1086208 PMC10203458

[56] Pollonini, L. et al. Auditory cortex activation to natural speech and simulated cochlear implant speech measured with functional near-infrared spectroscopy. *Hear. Res.*** 309**, 84–93 (2014). 24342740 10.1016/j.heares.2013.11.007 PMC3939048

[57] Peirce, J. W., Hirst, R. J. & MacAskill, M. R. Building Experiments in PsychoPy. *SAGE Publications Ltd. *https://uk.sagepub.com/en-gb/eur/building-experiments-in-psychopy/book273700 (2023).

[58] Strangman, G. E., Li, Z. & Zhang, Q. Depth Sensitivity and Source-Detector Separations for Near Infrared Spectroscopy Based on the Colin27 Brain Template. *PLoS One*** 8**, e66319 (2013). 23936292 10.1371/journal.pone.0066319 PMC3731322

[59] Rolls, E. T., Joliot, M. & Tzourio-Mazoyer, N. Implementation of a new parcellation of the orbitofrontal cortex in the automated anatomical labeling atlas. *NeuroImage*** 122**, 1–5 (2015). 26241684 10.1016/j.neuroimage.2015.07.075

[60] Tzourio-Mazoyer, N. et al. Automated Anatomical Labeling of Activations in SPM Using a Macroscopic Anatomical Parcellation of the MNI MRI Single-Subject Brain. *NeuroImage*** 15**, 273–289 (2002). 11771995 10.1006/nimg.2001.0978

[61] Brigadoi, S. & Cooper, R. J. How short is short? Optimum source–detector distance for short-separation channels in functional near-infrared spectroscopy. *Neurophotonics*** 2**, 025005 (2015). 26158009 10.1117/1.NPh.2.2.025005 PMC4478880

[62] Huppert, T. J. Commentary on the statistical properties of noise and its implication on general linear models in functional near-infrared spectroscopy. *Neurophotonics*** 3**, 010401 (2016). 26989756 10.1117/1.NPh.3.1.010401 PMC4773699

[63] Gramfort, A. et al. MEG and EEG data analysis with MNE-Python. *Front. Neurosci.*** 7**, 1–13 (2013). 24431986 10.3389/fnins.2013.00267 PMC3872725

[64] Luke, R. et al. Analysis methods for measuring passive auditory fNIRS responses generated by a block-design paradigm. *Neurophotonics*** 8**, 025008 (2021). 34036117 10.1117/1.NPh.8.2.025008 PMC8140612

[65] Abraham, A. et al. Machine learning for neuroimaging with scikit-learn. *Front. Neuoinformatics*** 8**, 14 (2014). 10.3389/fninf.2014.00014 PMC3930868 24600388

[66] Delpy, D. T. et al. Estimation of optical pathlength through tissue from direct time of flight measurement. *Phys. Med. Biol.*** 33**, 1433–1442 (1988). 3237772 10.1088/0031-9155/33/12/008

[67] Kocsis, L., Herman, P. & Eke, A. The modified Beer-Lambert law revisited. *Phys. Med. Biol.*** 51**, N91–N98 (2006). 16481677 10.1088/0031-9155/51/5/N02

[68] Santosa, H., Zhai, X., Fishburn, F. & Huppert, T. J. The NIRS Brain AnalyzIR Toolbox. *Algorithms*** 11**, 73 (2018). 38957522 10.3390/a11050073 PMC11218834

[69] Strangman, G. E., Franceschini, M. A. & Boas, D. A. Factors affecting the accuracy of near-infrared spectroscopy concentration calculations for focal changes in oxygenation parameters. *NeuroImage*** 18**, 865–879 (2003). 12725763 10.1016/s1053-8119(03)00021-1

[70] Glover, G. H. Deconvolution of impulse response in event-related BOLD fMRI. *NeuroImage*** 9**, 416 (1999). 10191170 10.1006/nimg.1998.0419

[71] R Core Team. R: A language and environment for statistical computing. R Foundation for Statistical Computing (2020).

[72] RStudio Team. RStudio: Integrated Development for R. RStudio, PBC (2020).

[73] Bates, D., Mächler, M., Bolker, B. M. & Walker, S. C. Fitting Linear Mixed-Effects Models Using lme4. *J. Stat. Softw. *(2015).

[74] Barr, D. J. Random effects structure for testing interactions in linear mixed-effects models. *Front. Psychol.*** 4**, 328 (2013). 23761778 10.3389/fpsyg.2013.00328 PMC3672519

[75] Bates, D., Kliegl, R., Vasishth, S. & Baayen, H. Parsimonious Mixed Models. Preprint at arXiv.1506.04967 (2015).

[76] Lenth, R. V. emmeans: Estimated marginal means, aka least-squares means [Computer software] (2021).

[77] Benjamini, Y. & Hochberg, Y. Controlling the false discovery rate: A practical and powerful approach to multiple testing. *J. R. Stat. Soc. Ser. B Methodol.*** 57**, 289–300 (1995).

