# Supplementary material for: Aesthetic evaluation of body movements shaped by embodied and arts experience: Insights from behaviour and fNIRS
Source: Sci Rep. 2024 Oct 28;14:25841. doi: 10.1038/s41598-024-75427-9 (PMC11519928; doi:10.1038/s41598-024-75427-9)
Supplement: Supplementary file 1 — Supplementary Information. [file 41598_2024_75427_MOESM1_ESM.pdf]

SUPPLEMENTARY MATERIALS - Aesthetic evaluation of body movements shaped by embodiment and arts experience: Insights from behaviour and fNIRS

STUDY 1

BEHAVIOURAL RESULTS (n = 41)

Table S1. Contrasts of behavioural ratings of movement sequences. SE = standard error; df = degrees of freedom; CI = confidence interval; statistical significance = $p < 0.05$.

	Time	Condition	ß	SE	df	95% CI	t ratio	p value
**Enjoyment**	Both	Known-Unknown	-8.83	0.91	1259	-11.11, -6.55	-9.69	< 0.001
	Post-Pre	Both	4.73	0.91	1259	2.46, 7.01	5.196	< 0.001
	Post	Known-Unknown	7.93	1.29	1259	4.71, 11.15	6.15	< 0.001
	Pre	Known-Unknown	1.54	1.29	1259	-1.68, 4.76	1.19	0.233
**Familiarity**	Both	Known-Unknown	-16.60	1.04	1264	-19.21, -14.00	-15.92	< 0.001
	Post-Pre	Both	10.84	1.04	1264	8.23, 13.44	10.39	< 0.001
	Post	Known-Unknown	22.53	1.47	1264	18.84, 26.22	15.28	< 0.001
	Pre	Known-Unknown	-0.86	1.47	1264	-4.55, 2.83	-0.58	0.561
**Reproducibility**	Both	Known-Unknown	-12.68	2.03	40	-17.99, -7.36	-6.24	< 0.001
	Post-Pre	Both	4.62	1.43	40	0.89, 8.35	3.24	0.003
	Post	Known-Unknown	9.48	1.77	94.2	4.96, 13.99	5.35	< 0.001
	Pre	Known-Unknown	-0.24	1.77	94.5	-4.76, 4.28	-0.13	0.894

INFLUENCE OF EMBODIMENT ON CORTICAL ACTIVITY – REPLICATION OF FMRI WORK

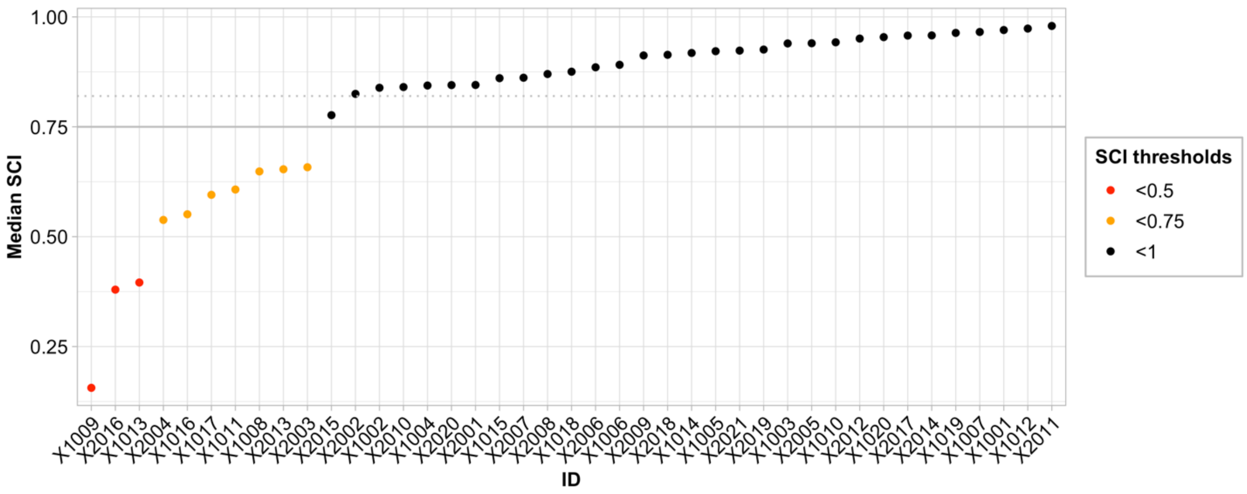

Figure S1. Median scalp-coupling index (SCI) per participant (ID) calculated per channel between frequencies between 0.7 and 1.35 Hz. SCI < 0.75 deemed to indicate poor signal quality, and accordingly, participants with SCI < 0.75 were excluded from group-level analyses.

Generation of waveforms for visual inspection. First, the time series values were converted from raw intensity to optical density. A scalp-coupling index (SCI) [1] was calculated per channel for frequencies between 0.7-1.35 Hz and channels with an SCI < 0.5 were excluded. To correct for motion artefacts, we used the temporal derivative distribution repair (TDDR) algorithm [2]. Using short-channel regression by the nearest short channel per long channel, we regressed out extracerebral and systemic components, to obtain signal components stemming from the cortex [3,4]. We next converted the signal from optical density to concentrations of HbO and HbR using the Modified Beer-Lambert Law [5,6]. We selected a partial pathlength factor of 0.1 to account for both the differential pathlength factor (DPF) and partial volume correction (PVC); (DPF = 6)/(PVC = 60) is equal to 0.1 [7,8]. Next, we implemented Cui et al.'s [9] algorithm, which capitalizes on the negatively correlated dynamics of HbO and HbR to enhance the signal-to-noise ratio. Next, we applied a bandpass-filter between 0.02-0.3 Hz to exclude slow drifts and cardiac components from the signal. We selected epoch from 3 s before stimulus onset to 30 s post-onset and linearly detrended the signal, accounting for slow drifts. Epochs with peak-to-peak differences >150 μM were excluded from the waveform plots.

Results–known vs. unknown. We fit separate models for HbO and HbR to the estimates from the GLMs with each the 6-s and 13-s boxcars: *HbO/HbR ~ 0 + ROI*condition + (1 + condition|ID)*. With the 6-s boxcar (i.e., the duration of video viewing) we observed significant reductions in HbO in left IFG, left MTG, right IFG for both known and unknown movements, as well as in right IPL for unknown movements. HbR was also significantly below zero in bilateral IFG and MTG for unknown movements (Figures S2A and B). With the 13-s boxcar, (i.e., including selection of response to the rating question), we observed significant increase in HbO in bilateral IPL for known and unknown movements. Additionally, HbR was significantly below zero in bilateral IPL, left MTG, and right IFG for known and unknown movements, and in left IFG for unknown movements (Figure S2A and C).

Next, we assessed differences in the cortical activation underlying the processing of known (embodied) and unknown movements using contrasts. For the 6-s-boxcar model, no differences between known and unknown movement sequences were observed in any ROI for HbO or HbR (*p*s all > .05, Table S2). For the 13-s boxcar model, known-unknown contrasts indicated a significant increase in HbR in left IFG only ($\beta = 1.29$, CI = [0.10, 2.47], $p = 0.025$), with no differences in any other ROIs (Table S3).

Brief discussion. Our findings from analyses of the video-viewing time-window (6-s boxcar) differ from past fMRI work, where embodied movement experience has been observed to increase cortical activation in AON regions when viewing and evaluating familiar movements [10,11]. We observed inverted haemodynamic responses in both HbO and HbR, where we expected a canonical haemodynamic response. Different methodologies may play a role in this discrepancy. Previous fMRI work has measured brain activity while participants view movement sequences passively, thereby measuring implicit aesthetic judgement, and tested explicit aesthetic ratings in a separate task outside of the scanner [11–13]. In our design, participants rated each sequence directly after viewing it, meaning that they were likely preparing an explicit aesthetic judgement while viewing. Based on this difference, we propose that the negative HbO and HbR estimates observed when applying the shorter 6-s boxcar function to trials that involved multiple components may highlight how viewing movement sequences as part of a more complex task can result in an overconsumption of HbO in the initial stages. Another possibility is that physiological events, such as blood pressure changes, muscle oxygenation or other extracerebral changes, may cause concurrent changes in both HbO and HbR [14,15]. Based on the nature of the task, it seems logical that our analyses of the whole trial (13-s

boxcar) are more consistent with Kirsch & Cross' [13] findings in young adults (matched to our sample). Kirsch & Cross [13] report significant increases in activation in bilateral angular gyri to be associated with enjoyment after dance training. We observed increased activation in ROIs covering bilateral IPL in our analyses of the whole visual and aesthetic rating time window (13-s boxcar). Given the differences in spatial resolution between fMRI and fNIRS, we propose that this pattern of activation is aligned with that reported by Kirsch & Cross' [13] for participants in the same age group.

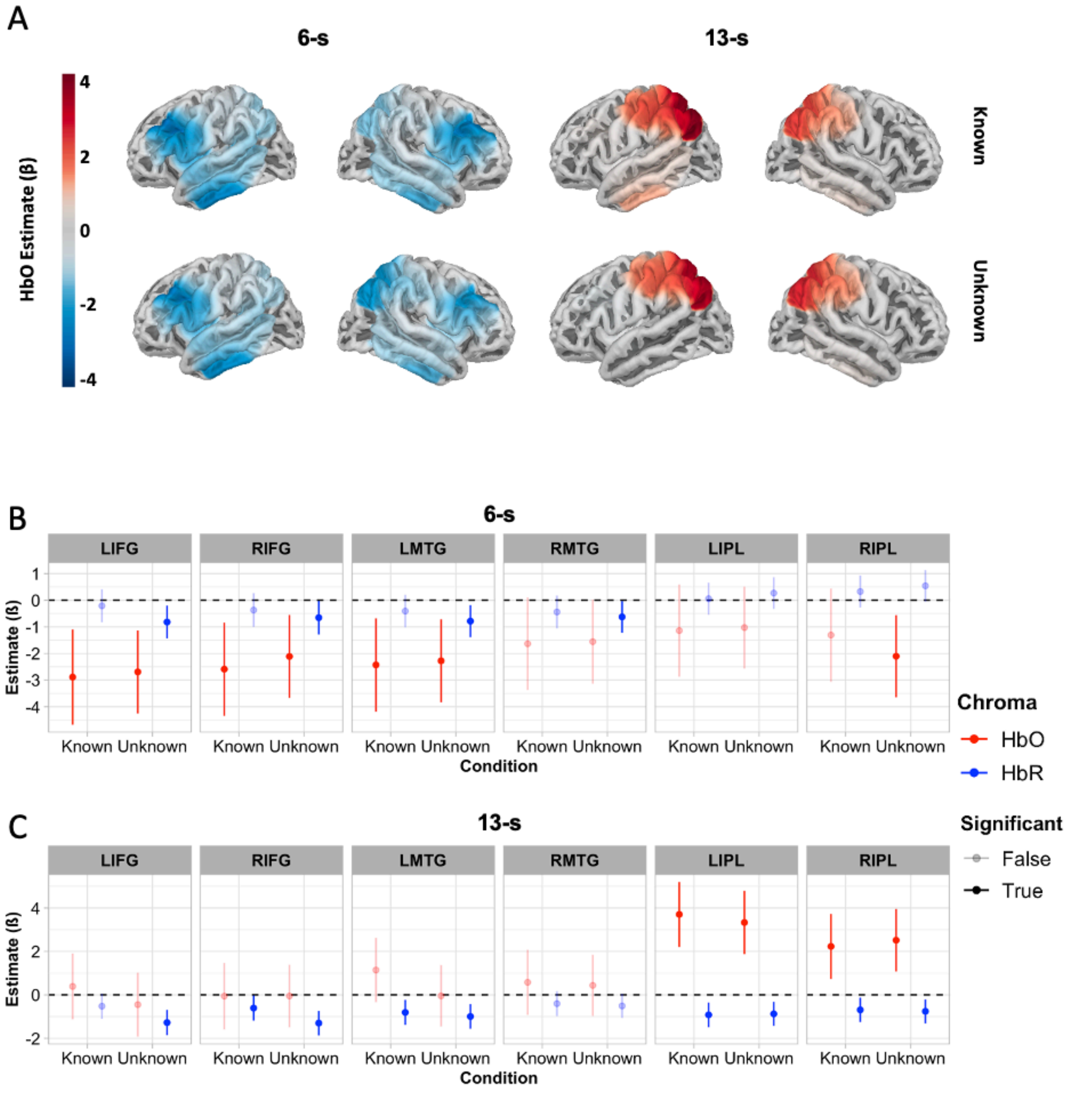

Figure S2. A) Estimates of cortical activation from GLM analysis projected onto brain surface (HbO only) using 6s- and 13-s boxcar model. B) 6-s boxcar (i.e., during video viewing), and C) 13-s boxcar (i.e., including aesthetic evaluation of stimulus). R = right, L = left, IFG = inferior frontal gyrus, MTG = middle temporal gyrus, IPL = inferior parietal lobule, HbO = oxygenated haemoglobin, HbR = deoxygenated haemoglobin.

Table S2. 6-s boxcar: Known-Unknown contrasts between estimates of cortical activation in each ROI. LIFG/RIFG = left/right interior frontal gyrus, LIPL/RIPL = left/right inferior parietal lobe, LMTG/RMTG = left/right middle temporal gyrus, Known = movements learned during training, Unknown = movements not learned during training, SE = standard error; df = degrees of freedom; CI = confidence interval; statistical significance = $p < 0.05$.

Chroma	ROI	ß	SE	df	95% CI	t ratio	p value
**HbO**	LIFG	-1.08	1.23	161	-4.36, 2.19	-0.88	0.860
	LIPL	0.35	1.23	161	-2.93, 3.62	0.28	0.860
	LMTG	0.22	1.23	161	-3.06, 3.49	0.18	0.860
	RIFG	-0.23	1.23	161	-3.51, 3.04	-0.19	0.860
	RIPL	0.97	1.23	161	-2.31, 4.24	0.79	0.860
	RMTG	1.30	1.23	161	-1.98, 4.57	1.06	0.860
**HbR**	LIFG	1.23	0.57	103	-0.32, 2.77	2.14	0.208
	LIPL	-0.21	0.57	103	-1.75, 1.33	-0.37	0.712
	LMTG	0.36	0.57	103	-1.18, 1.90	0.63	0.712
	RIFG	0.80	0.57	103	-0.74, 2.34	1.39	0.500
	RIPL	-0.21	0.57	103	-1.75, 1.33	-0.37	0.712
	RMTG	0.49	0.57	103	-1.05, 2.03	0.85	0.712

Table S3. 13-s boxcar: Known-Unknown contrasts between estimates of cortical activation in each ROI. LIFG/RIFG = left/right interior frontal gyrus, LIPL/RIPL = left/right inferior parietal lobe, LMTG/RMTG = left/right middle temporal gyrus, Known = movements learned during training, Unknown = movements not learned during training, SE = standard error; df = degrees of freedom; CI = confidence interval; statistical significance = $p < 0.05$.

Chroma	ROI	ß	SE	df	95% CI	t ratio	p value
**HbO**	LIFG	1.49	1.01	233	-1.19, 4.18	1.48	0.421
	LIPL	-0.22	1.01	233	-2.90, 2.47	-0.22	0.830
	LMTG	1.62	1.01	233	-1.06, 4.30	1.61	0.421
	RIFG	0.54	1.01	233	-2.15, 3.22	0.53	0.730
	RIPL	0.52	1.01	233	-2.17, 3.20	0.51	0.730
	RMTG	0.91	1.01	233	-1.78, 3.59	0.90	0.730
**HbR**	LIFG	1.29	0.45	330	0.10, 2.47	2.89	0.025
	LIPL	-0.14	0.45	330	-1.33, 1.04	-0.32	0.877
	LMTG	0.21	0.45	330	-0.97, 1.40	0.48	0.877
	RIFG	0.90	0.45	330	-0.29, 2.08	2.01	0.135
	RIPL	0.07	0.45	330	-1.12, 1.25	0.16	0.877
	RMTG	0.25	0.45	330	-0.94, 1.43	0.56	0.877

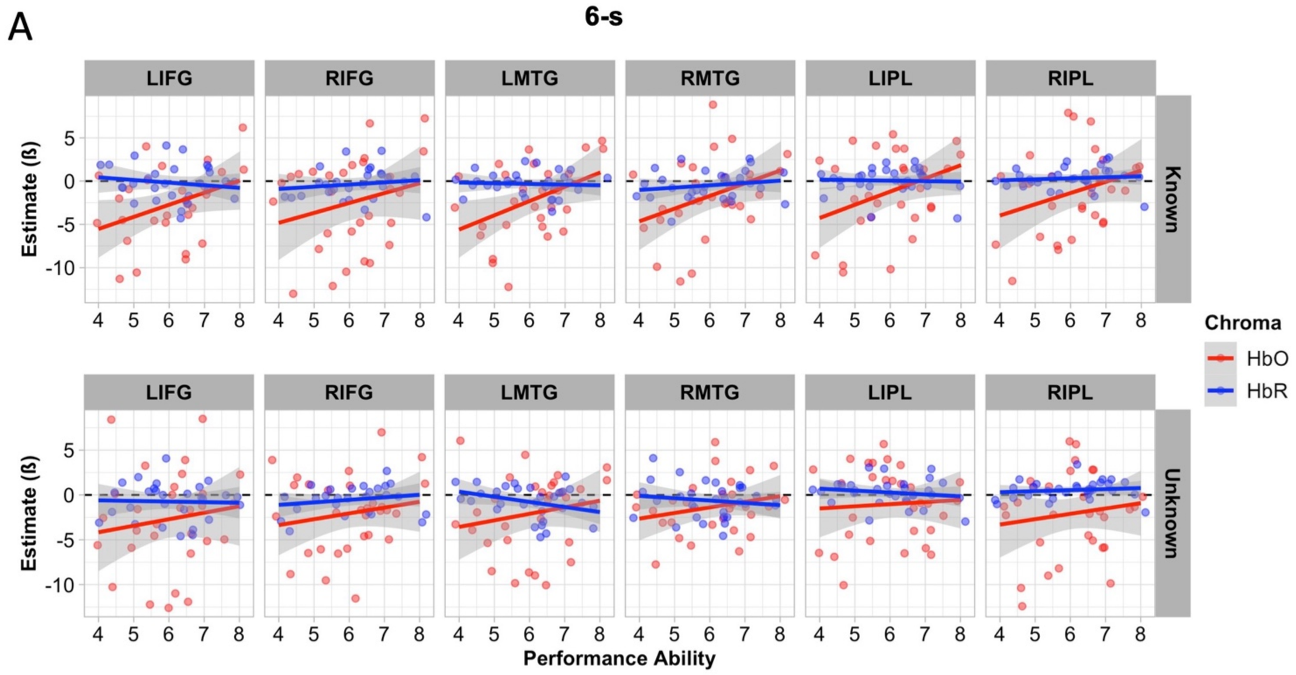

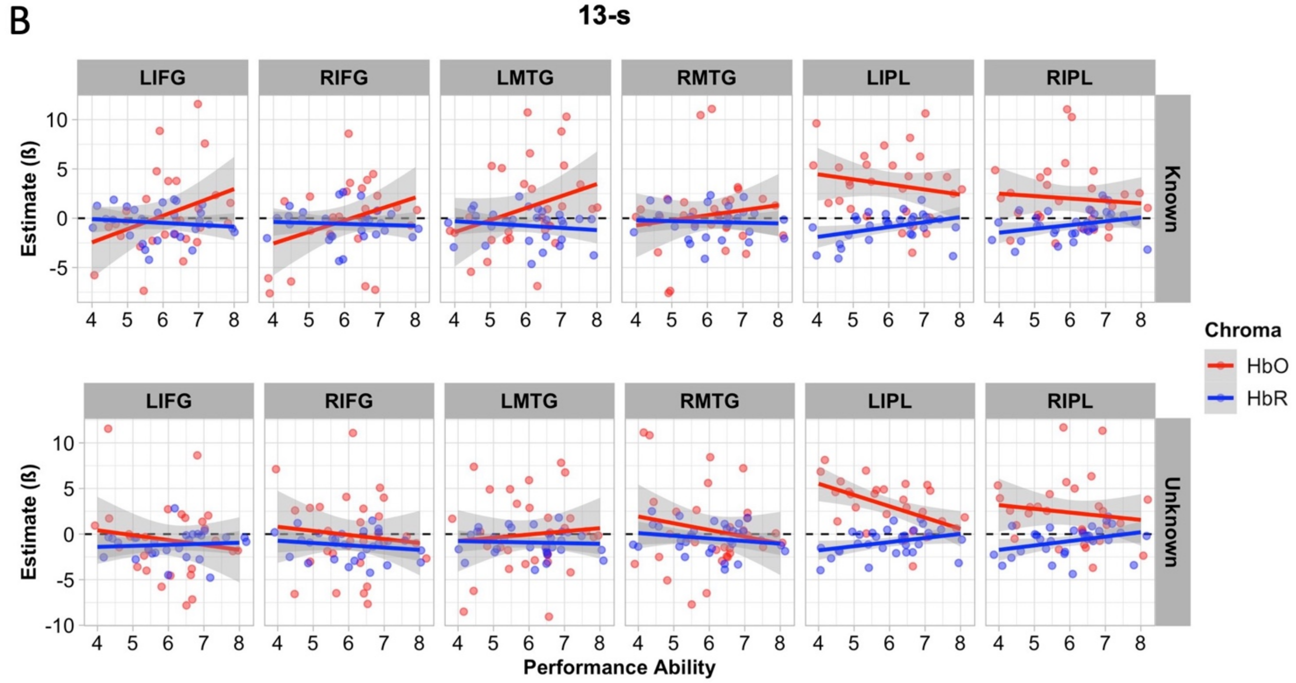

Figure S3. A) Relationship between performance ability and cortical activation while viewing videos (6-s boxcar) per ROI, for each HbO and HbR. B) Relationship between performance ability and cortical activation while viewing videos (13-s boxcar) per ROI, for each HbO and HbR.

Table S4. 6-s boxcar: Estimates of relationship between performance ability and cortical activity per ROI. LIFG/RIFG = left/right interior frontal gyrus, LIPL/RIPL = left/right inferior parietal lobe, LMTG/RMTG = left/right middle temporal gyrus, Known = movements learned during training, Unknown = movements not learned during training, SE = standard error; df = degrees of freedom; CI = confidence interval; statistical significance = p < 0.05.

Chroma	Condition	ROI	ß	SE	df	95% CI	t ratio	p value
**HbO**	Known	LIFG	1.95	1.00	79	-0.04, 3.95	1.95	0.055
		LIPL	1.52	1.00	79	-0.48, 3.52	1.51	0.134
		LMTG	2.58	1.00	79	0.59, 4.58	2.57	0.012
		RIFG	2.30	1.00	79	0.31, 4.30	2.30	0.024
		RIPL	1.72	1.00	79	-0.28, 3.72	1.72	0.090
		RMTG	2.03	1.00	79	0.03, 4.02	2.02	0.047
	Unknown	LIFG	1.12	0.83	144	-0.52, 2.77	1.35	0.179
		LIPL	0.24	0.83	144	-1.41, 1.89	0.29	0.773
		LMTG	1.24	0.83	144	-0.41, 2.89	1.49	0.139
		RIFG	0.84	0.83	144	-0.81, 2.48	1.01	0.317
		RIPL	0.60	0.83	144	-1.05, 2.24	0.72	0.475
		RMTG	0.82	0.83	144	-0.83, 2.47	0.99	0.326
**HbR**	Known	LIFG	0.45	0.40	85.3	-0.34, 1.24	1.14	0.259
		LIPL	-0.06	0.40	85.3	-0.85, 0.73	-0.15	0.883
		LMTG	-0.22	0.40	85.3	-1.01, 0.57	-0.55	0.585
		RIFG	0.67	0.40	85.3	-0.12, 1.45	1.68	0.097
		RIPL	0.12	0.40	85.3	-0.67, 0.90	0.29	0.772
		RMTG	0.33	0.40	85.3	-0.46, 1.12	0.84	0.405
	Unknown	LIFG	-0.01	0.37	106.7	-0.74, 0.72	-0.04	0.971
		LIPL	-0.22	0.37	106.7	-0.94, 0.51	-0.58	0.560
		LMTG	-0.67	0.37	106.7	-1.40, 0.05	-1.84	0.069
		RIFG	-0.01	0.37	106.7	-0.74, 0.72	-0.03	0.978
		RIPL	0.11	0.37	106.7	-0.61, 0.84	0.31	0.758
		RMTG	-0.26	0.37	106.7	-0.99, 0.47	-0.71	0.481

Table S5. 13-s boxcar: Estimates of relationship between performance ability and cortical activity per ROI. LIFG/RIFG = left/right interior frontal gyrus, LIPL/RIPL = left/right inferior parietal lobe, LMTG/RMTG = left/right middle temporal gyrus, Known = movements learned during training, Unknown = movements not learned during training, SE = standard error; df = degrees of freedom; CI = confidence interval; statistical significance = p < 0.05.

Chroma	Condition	ROI	ß	SE	df	95% CI	t ratio	p value
**HbO**	Known	LIFG	0.73	0.92	72.4	-1.11, 2.57	0.79	0.430
		LIPL	-0.52	0.92	72.4	-2.36, 1.32	-0.57	0.574
		LMTG	0.52	0.92	72.4	-1.32, 2.36	0.56	0.575
		RIFG	1.14	0.92	72.4	-0.70, 2.98	1.24	0.220
		RIPL	0.09	0.92	72.4	-1.75, 1.94	0.10	0.919
		RMTG	0.28	0.92	72.4	-1.56, 2.12	0.30	0.762
	Unknown	LIFG	-0.22	0.82	98.9	-1.84, 1.41	-0.26	0.793

		LIPL	-0.95	0.82	98.9	-2.58, 0.67	-1.16	0.248
		LMTG	0.35	0.82	98.9	-1.28, 1.97	0.42	0.673
		RIFG	-0.17	0.82	98.9	-1.80, 1.46	-0.21	0.835
		RIPL	-0.24	0.82	98.9	-1.86, 1.39	-0.29	0.774
		RMTG	-0.74	0.82	98.9	-2.36, 0.89	-0.90	0.371
**HbR**	Known	LIFG	0.01	0.34	200	-0.66, 0.68	0.03	0.975
		LIPL	0.56	0.34	200	-0.12, 1.23	1.63	0.105
		LMTG	-0.02	0.34	200	-0.69, 0.65	-0.06	0.954
		RIFG	0.04	0.34	200	-0.64, 0.71	0.11	0.915
		RIPL	0.39	0.34	200	-0.29, 1.06	1.13	0.260
		RMTG	0.15	0.34	200	-0.53, 0.82	0.44	0.664
	Unknown	LIFG	-0.29	0.34	200	-0.96, 0.38	-0.85	0.396
		LIPL	0.45	0.34	200	-0.22, 1.12	1.32	0.188
		LMTG	-0.14	0.34	200	-0.81, 0.53	-0.41	0.683
		RIFG	-0.06	0.34	200	-0.74, 0.61	-0.19	0.852
		RIPL	0.48	0.34	200	-0.19, 1.16	1.42	0.159
		RMTG	-0.29	0.34	200	-0.96, 0.39	-0.85	0.399

Table S6. Relationship between ability to perform learned choreography and aesthetic ratings of videos (known and unknown aggregated). SE = standard error; df = degrees of freedom; CI = confidence interval; statistical significance = $p < 0.05$.

	**ß**	**SE**	**df**	**95% CI**	**t ratio**	**p value**
**Enjoyment**	-1.15	1.16	39.1	-3.49, 1.19	-0.99	0.326
**Familiarity**	-2.77	1.43	39.1	-5.65, 0.12	-1.94	0.060
**Reproducibility**	-0.99	1.71	39.0	-4.45, 2.47	-0.58	0.567

STUDY 2

Table S7. Estimates of relationship between ratings of familiarity and reproducibility with choreography and experience with art forms which improved the model. SE = standard error; df = degrees of freedom; CI = confidence interval; statistical significance = $p < 0.05$.

	**Paint experience**	**ß**	**SE**	**df**	**95% CI**	**t ratio**	**p value**
**Familiarity**	No	61.8	1.72	138	58.5, 65.2	36.05	<0.001
	Yes	68.2	1.72	138	64.7, 71.7	38.88	<0.001
	**Dance experience**	**ß**	**SE**	**df**	**95% CI**	**t ratio**	**p value**
**Familiarity**	No	62.5	1.74	138	59.1, 66.0	35.87	<0.001
	Yes	67.5	1.73	138	64.1, 70.9	39.12	<0.001
	**Social media dance experience**	**ß**	**SE**	**df**	**95% CI**	**t ratio**	**p value**
**Reproducibility**	No	66.5	2.03	139	62.5, 70.5	32.79	<0.001
	Yes	73.2	1.65	139	70.0, 76.5	44.48	<0.001

Table S8. Relationship between reported enjoyment, familiarity and reproducibility and predictors retained in the best model. See methods for details regarding model selection. SE = standard error; df = degrees of freedom; CI = confidence interval; statistical significance = $p < 0.05$.

	**Art Form**	**ß**	**SE**	**df**	**95% CI**	**t ratio**	**p value**
**Enjoyment**	Theatre	0.09	0.05	137	-0.01, 0.19	1.70	0.092
	Dance	0.10	0.09	137	-0.07, 0.27	1.14	0.257
	Media Dance	0.12	0.07	137	-0.02, 0.25	1.71	0.091
**Familiarity**	Theatre	0.11	0.05	138	0.01, 0.21	2.28	0.024
	Media Dance	0.17	0.06	138	0.06, 0.27	2.98	0.003
**Reproducibility**	Music	0.26	0.12	138	0.01, 0.50	2.10	0.038
	Sports	0.11	0.05	137	0.00, 0.21	2.06	0.042
	Media Dance	0.11	0.06	137	-0.01, 0.22	1.78	0.077

References

1. Pollonini, L. *et al.* Auditory cortex activation to natural speech and simulated cochlear implant speech measured with functional near-infrared spectroscopy. *Hear. Res.* **309**, 84–93 (2014).
2. Fishburn, F. A., Ludlum, R. S., Vaidya, C. J. & Medvedev, A. V. Temporal Derivative Distribution Repair (TDDR): A motion correction method for fNIRS. *NeuroImage* **184**, 171–179 (2019).
3. Saager, R. B. & Berger, A. J. Direct characterization and removal of interfering absorption trends in two-layer turbid media. *J. Opt. Soc. Am. A Opt. Image Sci. Vis.* **22**, 1874–1882 (2005).
4. Scholkmann, F. *et al.* A review on continuous wave functional near-infrared spectroscopy and imaging instrumentation and methodology. *NeuroImage* **85**, 6–27 (2014).
5. Delpy, D. T. *et al.* Estimation of optical pathlength through tissue from direct time of flight measurement. *Phys. Med. Biol.* **33**, 1433–1442 (1988).
6. Kocsis, L., Herman, P. & Eke, A. The modified Beer-Lambert law revisited. *Phys. Med. Biol.* **51**, N91–N98 (2006).
7. Santosa, H., Zhai, X., Fishburn, F. & Huppert, T. J. The NIRS Brain AnalyzIR Toolbox. *Algorithms* **11**, 73 (2018).
8. Strangman, G. E., Franceschini, M. A. & Boas, D. A. Factors affecting the accuracy of near-infrared spectroscopy concentration calculations for focal changes in oxygenation parameters. *NeuroImage* **18**, 865–879 (2003).
9. Cui, X., Bray, S. & Reiss, A. L. Functional near infrared spectroscopy (NIRS) signal improvement based on negative correlation between oxygenated and deoxygenated hemoglobin dynamics. *NeuroImage* **49**, 3039–3046 (2010).
10. Cross, E. S., Kraemer, D. J. M., Hamilton, A. F. d. C., Kelley, W. M. & Grafton, S. T. Sensitivity of the Action Observation Network to Physical and Observational Learning. *Cereb. Cortex* **19**, 315–326 (2009).
11. Kirsch, L. P., Dawson, K. & Cross, E. S. Dance experience sculpts aesthetic perception and related brain circuits. *Ann. N. Y. Acad. Sci.* **1337**, 130–139 (2015).
12. Calvo-Merino, B., Jola, C., Glaser, D. E. & Haggard, P. Towards a sensorimotor aesthetics of performing art. *Conscious. Cogn.* **17**, 911–922 (2008).
13. Kirsch, L. P. & Cross, E. S. The influence of sensorimotor experience on the aesthetic evaluation of dance across the life span. in *Progress in Brain Research* vol. 237 291–316 (Elsevier, 2018).
14. Yücel, M. A. *et al.* Best practices for fNIRS publications. *Neurophotonics* **8**, 012101 (2021).
15. Zimeo Morais, G. A., Balardin, J. B. & Sato, J. R. fNIRS Optodes' Location Decider (fOLD): a toolbox for probe arrangement guided by brain regions-of-interest. *Sci. Rep.* **8**, 3341 (2018).
